# Racial disparities in healthcare-associated infections: a systematic review and meta-analysis

**DOI:** 10.1017/ice.2026.10461

**Published:** 2026-06

**Authors:** Reinaldo Perez, Sonali D. Advani, Rebecca North, Alison G.C. Smith, Ibukunoluwa C. Kalu, Steph Hendren, Sarah Peskoe, Carl Pieper, Helen Zhang, Melissa Campbell, Sophie Nick, Michael Yarrington, Erin Gettler, Jay Krishnan, Nwora Lance Okeke, Janine Young, Deverick J. Anderson

**Affiliations:** 1 Duke University, USA; 2 Duke Center for Antimicrobial Stewardship and Infection Prevention, Durham, NC, USA; 3 University of California San Diego, USA

## Abstract

**Objective:**

Racial and ethnic disparities in healthcare-associated infections (HAIs) could have significant implications for hospital-based incentive programs. We sought to quantify racial and ethnic disparities in HAIs across inpatients in the United States.

**Design:**

Systematic Review and Meta-Analysis.

**Methods:**

Medline, Embase, and Scopus were searched 2008–2022 for English language studies describing reportable HAIs among inpatients and references to race or ethnicity. Studies were excluded if they used an aggregate outcome of “infection,” described a non-acute care setting, or measured a respiratory virus. Meta-analysis was performed using random-effects model on pooled continuous outcomes with 3 or more studies.

**Results:**

39 studies met criteria for inclusion; 23 evaluated surgical site infections (SSI), 6 evaluated hospital-onset *Clostridioides difficile* infection (HO-CDI), 5 evaluated central line-associated bloodstream infections (CLABSI), and 8 examined other HAIs. There was a high degree of heterogeneity across studies. Meta-analysis was performed for 10 distinct HAI/risk factor combinations. Race and ethnicity were not associated with SSIs (Black vs White OR 1.19, 95% CI 0.95–1.48; Hispanic vs White OR 1.01, 95% CI 0.78–1.31). Hispanic patients had lower risk of HO-CDI relative to White patients (OR 0.80, 95% CI 0.64–0.98). Black (OR 1.36, 95% CI 1.07–1.70) or Hispanic (OR 1.16, 95% CI 1.073–1.249) patients had increased risk of CLABSI compared with White patients.

**Conclusions:**

Racial and ethnic disparities were observed in rates of HAIs, specifically for CLABSI and HO-CDI. However, conclusions are limited by the substantial heterogeneity present. Further research characterizing social determinants of health driving these disparities is needed.

## Introduction

The Centers for Disease Control and Prevention (CDC) estimates that approximately 1 in 31 hospitalized patients has at least one healthcare-associated infection (HAI).^1^ As hospitals seek to reduce and eliminate HAIs, a key step is understanding the causes of health disparities.^2^ Health disparities are health differences linked to economic, social and/or environmental disadvantages. These disparities are best captured using the framework of social determinants of health (SDOH), or “the conditions in which people are born, grow, live, work and age.”^3^ Attention is shifting towards identifying and addressing disparities in HAIs. Evaluation of health disparities data and the association with HAIs may have significant implications for quality improvement metrics and hospital reimbursement.^4^

Although there is increasing interest in racial and ethnic disparities in HAIs and associations with SDOH, the scope of evidence on the topic has not been widely reported.^5^ A number of studies have looked at race, ethnicity, and/or SDOH and associations with specific HAIs, but few studies have summarized the literature sufficiently to inform national policy.^6–8^ To better understand the current landscape, we performed a systematic review and meta-analysis to evaluate the disparities in HAIs by race and ethnicity with additional review for SDOH measures. The review is centered around racial and ethnic disparities, but the authors recognize the differential healthcare exposures and structural inequities which drive these disparities.^9^ We hypothesized that we would find significant differences in the rates of HAIs across different racial and ethnic groups.

## Methods

**Systematic Review Methodology:** We followed the 2020 Preferred Reporting Items for Systematic reviews and Meta-Analyses (PRISMA) checklist to ensure that all components of preferred methodology were incorporated into this review. Review protocol was registered with the PROSPERO database (Protocol# CRD42024429721) and the full search strategy with inclusion/exclusion criteria are available in the supplement (S1–S3). The primary review question was: Is there an association between race, ethnicity and social determinants of health and the incidence of HAIs? We searched Medline (OVID), Embase (Elsevier), and Scopus (Elsevier) for manuscripts published between 2008 and 2022 describing reportable HAIs among inpatients and with reference to race or ethnicity. We imported all citations to Covidence (www.covidence.org, Veritas Health Innovation) and removed duplicates. Studies were included if differences in HAIs based upon race, ethnicity or SDOH were evaluated. We used the Office of Disease Prevention and Health Promotion’s Healthy People 2030 framework for SDOH with 5 major categories: economic stability, neighborhood and built environment, education access and quality, social and community context, and health care access and quality.^3^ Given the importance of social context, we only included studies based in the United States. Two blinded reviewers (listed authors) assessed all studies first in a title and abstract review. If both reviewers agreed that the study qualified, the article moved to a full text review. Studies undergoing full text review were again assessed by two blinded reviewers and required agreement to be included in the final review. At both stages, discrepancies between reviewers were resolved by a third blinded reviewer. Studies under consideration were then evaluated utilizing the Joanna Briggs Institute critical appraisal tools for an assessment of bias and internal validity before final inclusion (S4).^10^ All studies that met these inclusion criteria were summarized as part of the systematic review. Relevant statistical data were extracted from the studies into a secure REDCap database for incorporation into the accompanying meta-analysis.

### Statistical analysis

Meta-analysis was employed for data synthesis when at least three manuscripts identified by the systematic review examined a common infection, common exposure, and common reference group (e.g., rate of HAI in Black vs White patients) and provided sufficient statistical information (e.g., odds ratios). Among studies that met these criteria, infections evaluated included surgical site infection (SSI), central line-associated bloodstream infection (CLABSI), and hospital-onset *Clostridioides* difficile (HO-CDI). No meta-analysis was performed related to methicillin-resistant *Staphylococcus aureus* (MRSA) infections, ventilator-associated pneumonias or other HAI outcomes due to inadequate numbers of studies. Exposure/reference categories that were considered included race, ethnicity, sex, and insurance status with reference categories based upon source literature. Unadjusted log relative risks (RR) or unadjusted log odds ratios (OR) from the individual studies were combined using random-effects meta-analysis, with heterogeneity estimated by previously established methods.^11–13^ If RR or OR were directly reported in the individual studies, standard errors were extracted from the study or derived from the 95% confidence intervals. If RR or OR were not directly reported, but sample sizes and event counts were reported, then the metrics and corresponding standard errors were calculated. The final RR or OR, 95% confidence interval, and test of heterogeneity were reported. SAS version 9.4 (SAS Institute, Inc., Cary, NC, USA) was used for all analyses.

## Results

**Figure 1.**
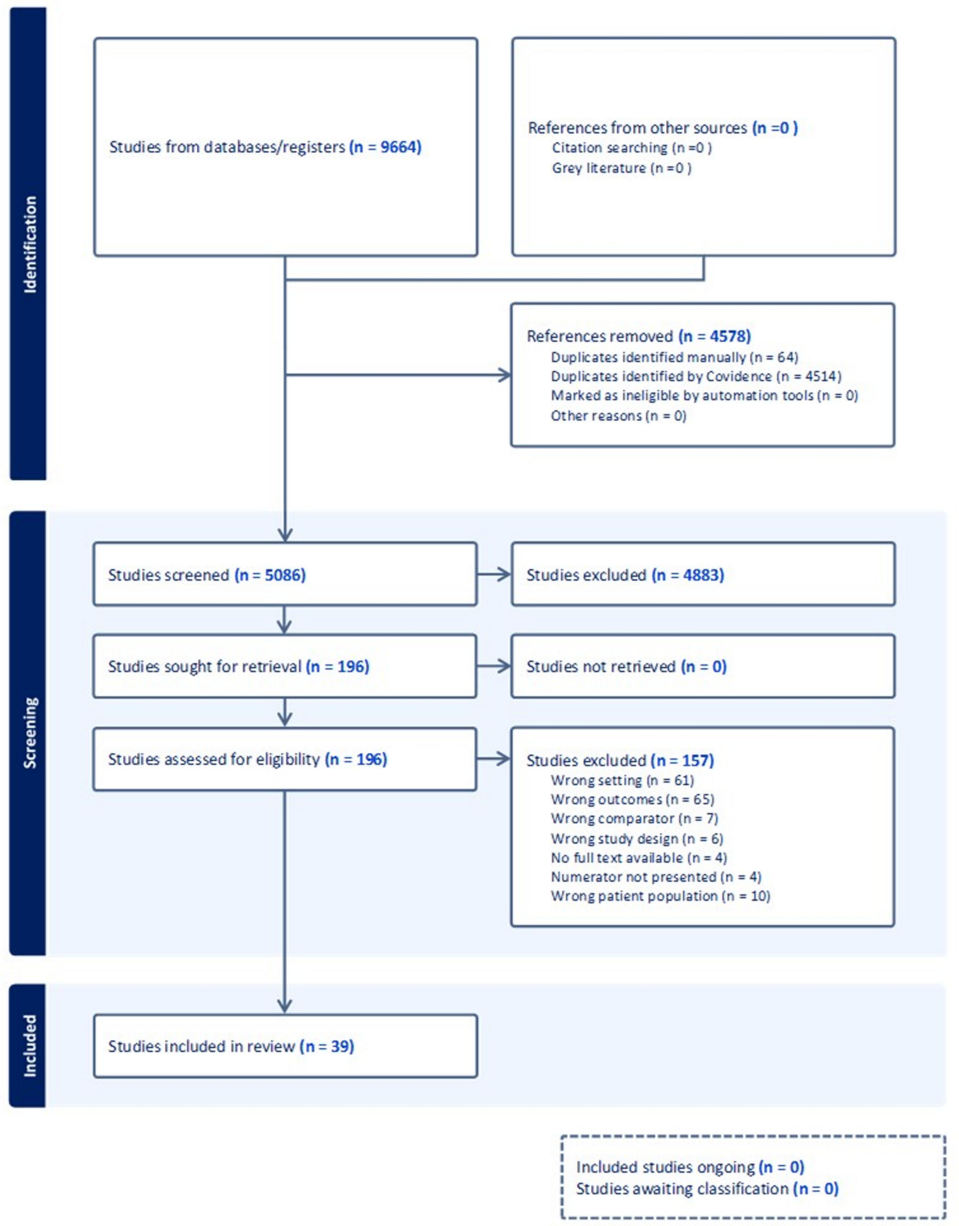
Figure 1 long description. PRISMA diagram for study inclusion.

We screened 5,150 titles and abstracts; 4,883 studies were excluded due to irrelevance, leaving 203 studies for full text review. Of these, 39 studies met criteria for inclusion in our systematic review (Figure 1). Details for each included study are summarized in Table 1. Included studies primarily consisted of retrospective cohort data with the addition of two prospective cohort studies and two retrospective case-control studies. Of these 39 studies,^7,8,14–50^ 18 prespecified an investigation into race, ethnicity or SDOH,^7,8,15–17,20,27,30–34,38–40,44,46,49,50^ while the remaining studies broadly assessed patient characteristics and reported a result relevant to our systematic review. Twenty-three studies examined SSI,^7,14–35^ 6 examined CDI,^7,40–44^ 5 examined CLABSI,^7,36–39^ and 8 explored other HAIs or pooled infection outcomes^7,8,45–50^ (Table 1). Seven studies focused on pediatric patients^18,29,38,39,45,47,48^ and the remaining 32 analyzed adults. The most commonly studied exposures were race and ethnicity (*n* = 32), followed by age (*n* = 16), sex (*n* = 15), and insurance status (*n* = 11). Based on the available data, 22 studies were included in the summary meta-analyses.^7,16,19–31,35,38–40,42,44,49^ There was sufficient data to examine 10 HAI/exposure combinations with meta-analytic techniques. A visual summary of included study findings by HAI and exposure is available in the supplement (S5,S6).

**Table 1.** Included studies by healthcare-associated infection (HAI) including study designs and results Table 1 long description.

First author (year) included in statistical analysis (yes/no)	Population and setting N	Geography	Outcome	Design and data	Health equity focus prespecified	Equity markers	Results
Surgical site infection							
Allareddy (2015) **No**	Adults hospitalized and undergoing a major surgical procedure N = 22,932,947	Across United States	MRSA infection (SSI, HO-MRSA Bacteremia, and MRSA pneumonia)	Retrospective cohort, 2009–2010, US Nationwide Inpatient Sample	No—Study broadly assessed characteristics	Race, ethnicity, age sex, health insurance status	Increased Risk: Increasing age per 1-year increment (aOR 1.0034, 95% CI 1.0023–1.0045), Black race (aOR 1.19, 95% CI 1.13–1.24), Native American race (aOR 1.27, 95% CI 1.01–1.6) Decreased Risk: Female sex (aOR 0.68, 95% CI 0.66–0.70), Medicare vs uninsured (aOR 0.63, 95% CI 0.59–0.68) Medicaid vs uninsured (aOR 0.71, 95% CI 0.67–0.76) Private insurance vs uninsured (aOR 0.47, 95% CI 0.44–0.5) Hispanic ethnicity (aOR 0.85, 95% CI 0.79–0.91) Asian/Pacific Islander Race (aOR 0.58, 95% CI 0.52–0.65)
Arsoniadis (2017) **No**	Adults with Crohn’s disease undergoing bowel surgery N = 9,513	Across United States	SSI	Retrospective cohort, 2005–2013, American College of Surgeons National Surgical Quality Improvement Program (ACS-NSQIP)	Yes	Race	Increased risk: Black race (No effect estimate given, *P* value only, .037)
Bakullari (2014) **Yes**	Adults hospitalized with acute cardiovascular disease, pneumonia, or major surgery N = 79,019	Across United States	Pooled HAI event (HO-CDI, CLABSI, CAUTI, HO-MRSA, HO-PNA, SSI)	Retrospective cohort, 2009–2011, Medicare Patient Safety Monitoring System	Yes	Race, ethnicity	Increased risk: Hispanic ethnicity (aOR 1.3 95% CI 1.15–1.53), Asian race (aOR 1.4 95% CI 1.07–1.75) No change: Black Race (aOR 1.1 95%CI 0.99–1.23), Native Hawaiian/ Pacific Islander (aOR 0.7, 95% CI 0.4–1.12)
Blum (2013) **Yes**	Adults undergoing primary elective total knee arthroplasty N = 17,385	Pennsylvania	SSI	Retrospective cohort, 2001–2007, Pennsylvania Health Care Cost Containment Council database	Yes	Race	No difference seen across races (no OR given just *P* value of .77 for comparison of Black and White patients)
Browne (2014) **No**	Adults undergoing elective total knee or total hip arthroplasty N = 214,217	Across United States	SSI	Retrospective Cohort, 2002–2011, Nationwide Inpatient Sample	Yes	Insurance status	Increased risk: Medicaid insurance (ref: “non-Medicaid insurance”) aOR 1.7 (95% CI 1.3–2.1)
Bucher (2011) **Yes**	Children (Age 0–18) undergoing general surgery, orthopedic surgery and plastic surgery N = 477	Missouri	SSI	Retrospective Cohort, 1996–2008, St. Louis Children’s Hospital records	No—Study broadly assessed characteristics	Race, age	Increased Risk: Black race (aOR 2.38, 95% CI 1.33–4.18) neonates < 30 days old (aOR 4.97 95% CI 1.37–17.9, ref adolescent > 12yr) No difference: Infant > 30 days < 1 year (aOR 1.7 95% CI 0.67–4.36, ref adolescent > 12yr) Child 1yr to 12yr (aOR 0.92, 95% CI 0.43–1.94, ref adolescent > 12 yr)
Chen (2019) **Yes**	Women undergoing Hysterectomy N = 125,337	United States and Canada	SSI	Retrospective cohort, 2006–2015, American College of Surgeons National Surgical Quality Improvement Program (ACS-NSQIP)	No—Study broadly assessed characteristics	Race, age	Decreased risk: Age > 40 (aOR 0.77 95% CI 0.65–0.82, ref age < 40) No change: Black race (aOR 0.92 95% CI 0.83–1.03), “Other” race (aOR 1.03, 95% CI 0.86–1.22)
Edwards (2020) **Yes**	Adults undergoing robotic assisted revisional metabolic and bariatric surgery N = 1,922	Across United States	SSI	Retrospective cohort, 2015–2017, Metabolic and Bariatric Surgery Accreditation and Quality Improvement Database	Yes	Race	No change: Black race (*P* = .4, no effect estimate given Hispanic ethnicity (*P* = 1, no effect estimate given)
Hogle (2014) **Yes**	Adults undergoing cardiac surgery N = 3,418	New York	SSI	Retrospective cohort, 2006–2008, Columbia University data set	No—Study broadly assessed characteristics	Race	No difference seen across races Hispanic ethnicity (aOR 1.14, 95% CI 0.56–2.3) Black race (aOR 0.78, 95% CI 0.31–1.95) “Other” race (aOR 1.04, 95% CI 0.55–1.95)
Liang (2013) **Yes**	Adults undergoing ileostomy or colostomy reversal N = 128	Texas	SSI	Retrospective cohort, 2005–2011, Houston Veterans Affairs Medical Center Data	No—Study broadly assessed characteristics	Race, age, sex, obesity	Increased Risk: Obesity assessed by CT scan (aOR 2.02, 95% CI 1.33–3.21) Decreased risk: Black race (aOR 0.35, 0.13–0.86) No change: age, sex (no effect estimates given)
Myssiorek (2018) **Yes**	Adults undergoing thyroidectomy N = 57,371	Across United States	SSI	Retrospective cohort, 2012–2015, American College of Surgeons National Surgical Quality Improvement Program (ACS-NSQIP)	No—Study broadly assessed characteristics	Race, age, sex	Increased risk: Age > 80 (aOR 2.41, 1.14–5.07) Male sex (aOR 2.04 95% CI 1.46–2.84) No change: Black race (aOR 1.45, 95% CI 0.95–2.21)
Namba (2012) **Yes**	Adults undergoing primary elective total hip arthroplasty N = 30,491	Across United States	SSI	Prospective cohort, 2001–2009, Kaiser Permanente Total Joint Replacement Registry	No—Study broadly assessed characteristics	Race, age, sex, BMI	Increased risk: BMI > 35 (aOR 2.37, 95% CI 1.55–3.61) Decreased risk: Female sex (aOR 0.7, 95% CI 0.49–0.99) No Change: BMI < 18.5 (aOR 2.88 95% CI 0.89–9.23) Black or Hispanic (no effect estimate given)
Namba (2013) **Yes**	Adults undergoing primary elective total knee arthroplasty N = 56,216	Across United States	SSI	Prospective cohort, 2001–2009, Kaiser Permanente Total Joint Replacement Registry	No—Study broadly assessed characteristics	Race, age, sex, BMI	Increased Risk: Male sex (aOR 1.89, 95% CI 1.54–2.32), BMI > 35 (aOR 1.47, 95% CI 1.17–1.85) Decreased Risk: Hispanic ethnicity (aOR 0.69, 95% CI 0.49–0.98), No change: Age (no effect estimate), Black race (aOR 1.23, 95% CI 0.88–1.62), Asian race (aOR 0.99, 95% CI 0.6–1.62)
Poultsides (2013) **Yes**	Adults undergoing primary elective total hip or total knee arthroplasty N = 1,196,691	Across United States	SSI	Retrospective cohort, 1998–2007, Nationwide Inpatient Sample	No—Study broadly assessed characteristics	Race, sex, age	Increased risk: Age < 44 (ref 45–65) (aOR 1.7 95% CI 1.48–1.95) Male sex (aOR 1.33, 95% CI 1.25–1.42) Black race (aOR 1.39, 95% CI 1.22–1.58) Hispanic ethnicity (aOR 1.5, 95% CI 1.28–1.75)Decreased risk: Age 65–74 (aOR 0.9, 95% CI 0.83–0.97) No change: Age 75 + (aOR 0.98, 95% CI 0.9–1.06)
Qi (2019) **Yes**	Adults undergoing colectomy or abdominal hysterectomy Colectomy N = 90,210 Hysterectomy N = 59,531	Arizona, Florida, Iowa, Maryland, Massachusetts, New York, Vermont	SSI	Retrospective cohort, 2013–2014 State inpatient databases of 9 delineated states	Yes	Race, insurance status, neighborhood income	**For Colectomy:** Increased risk: Medicare (ref private) (aOR 1.25, 95% CI 1.1–1.41) Medicaid (ref: private) (aOR 1.23, 95% CI 1.06–1.44) bottom quartile income (ref: top quartile) (aOR 1.14, 95% CI 1.01–1.29) Decreased risk: Black race (aOR 0.71, 95% CI 0.61–0.82) No change: Hispanic ethnicity (aOR 0.89, 95% CI 0.76–1.06) For Hysterectomy: Increased risk: Medicare insurance (ref: private) (aOR 1.47, 95% CI 1.01–2.13) No change: Black race (aOR 1.26, 95% CI 0.96–1.66) Hispanic ethnicity (aOR 0.86, 95% CI 0.6–1.24), Medicaid insurance(ref: private) (aOR 1.27, 95% CI 0.95–1.7), income quartile (aOR 1, 95% CI 0.73–1.39)
Richards (2014) **No**	Adults undergoing primary total shoulder arthroplasty N = 3,906	California	SSI	Retrospective cohort, 2005–2011, Kaiser Permanente data set	No—Study broadly assessed characteristics	Age, sex	Increased risk: Male sex (aOR 2.59, 95% CI 1.27–5.31) Decreased risk: increasing age (per year increment) (aOR 0.95, 95% CI 0.92–0.98)
Simon (2009) **Yes**	Children (Age 0–18) undergoing initial CSF Shunt placement N = 7,071	Across United States	SSI	Retrospective cohort, 2001–2005, Pediatric Health Information System database	No—Study broadly assessed characteristics	Race, age, sex	Increased risk: Age < 48 months (ref > 48 months) (aOR 1.6, 95% CI 1.2–2.1), female sex (aOR 1.2, 95% CI 1–1.4) Black race (aOR 1.5, 95% CI 1.2–1.9), Asian race (aOR 1.7, 95% CI 1.0–3.0) Medicaid insurance (relative to private) (aOR 1.3, 95% CI 1.1–1.6) No change: Latino race (aOR 0.9, 95% 0.7–1.1)
Singh (2020) **Yes**	Adults undergoing total ankle arthroplasty N = 6,280	Across United States	SSI	Retrospective cohort, 1998–2014 US National Inpatient Sample	Yes	Race, age, insurance status	Decreased risk: Age 51–80 (ref <50) (aOR 0.07, 95% CI 0.01–0.43), Black race (aOR < 0.01, 95% CI < 0.01– <0.01) No change: Sex (aOR 0.13, 95% CI 0.01–1.89), Hispanic ethnicity (aOR 9.3, 95% CI 1.27–68.05), Medicare (relative to private insurance) (aOR 2.25, 95% CI 0.63–8.01), Income percentile (aOR 2.86, 95% CI 0.3–27.33)
Singh (2022) **Yes**	Adults undergoing tympanoplasty N = 11,701	Across United States	SSI	Retrospective Cohort 2005–2019, National Surgical Quality Improvement Program database	Yes	Race	Increased risk: Black race (aOR 6.29, 95% CI 1.6–24.7) “other” race (Not Black Hispanic, Asian or American Indian) (aOR 10.92, 95% CI 1.16–84.22) No change: Asian race (aOR 1.05, 95% CI 0.13–8.37) Hispanic ethnicity (aOR 0.981, 95% 0.11–8.16)
Theiss (2022) **No**	Adult patients undergoing elective colorectal surgery N = 552	Alabama	Pooled surgical complication (including SSI)	Retrospective cohort, 2015–2020, University of Alabama at Birmingham database	Yes	Race, literacy	Increased risk: Black race (aOR 1.64, 95% CI 1.05–2.57), limited health literacy (aOR 2.03, 95% CI 1.01–3.08)
Trilles (2022) **No**	Adult patients undergoing gender affirming surgery N = 2,308	Across United States	SSI Readmission	Retrospective Cohort, 2010–2018, American College of Surgeons National Surgical Quality Improvement Program (ACS-NSQIP)	Yes	Race	**SSI:** Increased risk: Black race (aOR 4.65, 95% CI 1.22–17.67) **Readmission:** Increased risk: Black race (aOR 2.46, 95% CI 1.35–4.47) No change: Asian race (aOR 1.05, 0.25–4.48)
Wang (2022) **No**	Adults over age 65 undergoing primary total knee or total hip arthroplasty N = 697,317	Across United States	SSI	Retrospective cohort 2015–2020, Premier National Database	Yes	Health insurance status	Increased risk: Medicare Advantage plans (relative to traditional Medicare) (aOR 1.15, 1.04–1.47)
Zhao (2014) **Yes**	Adults undergoing genital reconstruction surgery N = 82	Michigan	SSI	Retrospective cohort, 1984–2008, Harper University Hospital data set	No—Study broadly assessed characteristics	Race	No difference seen between black and white races (aOR 0.22, 95% CI 0.04–1.2)
Central Line Associate Bloodstream Infection							
Bakullari (2014) **Yes**	Adults hospitalized with acute cardiovascular disease, pneumonia, or major surgery N = 79,019	Across United States	CLABSI (Subset)	Retrospective cohort, 2009–2011, Medicare Patient Safety Monitoring System	Yes	Race, ethnicity	No change: Black Race (aOR 1.34 95%CI 0.73–2.44)
Fargen (2015) **No**	Adults hospitalized for acute ischemic stroke N = 1,507,336	Across United States	CAUTI CLABSI	Retrospective cohort, 2002–2011, Nationwide Inpatient Sample	No—Study broadly assessed characteristics	Insurance status	No change: Medicare and Medicaid insurance (ref private) (aOR 0.89, 95% CI 0.89–1.01)
Gouel-Cheron (2022) **No**	Adults hospitalized in ICU N = 2,529,158	Across United States	ICU onset BSI	Retrospective cohort 2009–2015, Cerner Health Facts Database	No—Study broadly assessed characteristics	Race, ethnicity, age sex, healthcare facility origin	Increased Risk: Black race (HR 1.35, 95% CI 1.17–1.55) Hispanic Ethnicity (HR 1.9, 95% CI 1.26–2.87) Decreased risk: Younger Age (HR 0.93, 95% CI 0.89–0.96) Female sex (HR 0.82, 95% CI 0.73–0.92) No change: Healthcare facility origin (HR 1.14, 95% CI 0.97–1.35)
Snyder (2020) **Yes**	Neonates with gastroschisis N = 2,032	Across United States	CLABSI	Retrospective cohort, 2016, Healthcare Cost and Utilization Project Kid’s Inpatient Database	Yes	Race, geography	Increased risk: Black race (RR 2.17, 95% CI 1.14–4.15), suburban regions (ref, large metropolitan, po*P* > 1 million) (No effect estimate given), Southern US (ref: northeast) (No effect estimate given) No change: Hispanic ethnicity (RR 1.15, 95% CI 0.88–2.6) “Other” race (RR 0.73, 95% CI 0.36–1.46), US west and Midwest, rural and small metro areas
Willer (2022) **Yes**	Children (age < 18) admitted and requiring CVC placement N = 226,802	Across United States	CLABSI	Retrospective cohort, 2016–2021, Pediatric Health Information System database	Yes	Race	Increased risk: Black race (RR 1.27, 95% CI 1.17–1.37) Hispanic ethnicity (RR 1.16, 95% CI 1.08–1.26) No change: “other” race (RR 1.09, 0.99–1.21)
*Clostridioides difficile* Infection							
Argamany (2016) **Yes**	Adults hospitalized with CDI N = 1,676,903	Across United States	CDI (all not just HO-CDI)	Retrospective cohort, 2001–2010, US National Hospital Discharge Survey	Yes	Race	Increased risk: Black race (aOR 1.09, 95% CI 1.07–1.11)
Bakullari (2014) **Yes**	Adults hospitalized with acute cardiovascular disease, pneumonia, or major surgery N = 79,019	Across United States	CDI Subset	Retrospective cohort, 2009–2011, Medicare Patient Safety Monitoring System	Yes	Race, ethnicity	No change: Hispanic ethnicity (aOR 1.11, 95% CI 0.686–1.796)
Egorova (2015) **No**	Adults undergoing major vascular surgery N = 450,325	Across United States	HO-CDI	Retrospective cohort, 2000–2011, Nationwide Inpatient Sample	No—Study broadly assessed characteristics	sex, insurance, rurality	Increased risk: Medicare or Medicaid insurance (*P* < .001, no effect estimate given) Decreased risk: rural hospital (*P* < .001, no effect estimate given) No change: sex (*P* = .12, no effect estimate)
Rosenblatt (2019) **Yes**	Adults admitted with advanced cirrhosis N = 3,104,310	Across United States	CDI (all not just HO-CDI)	Retrospective cohort, 1998–2014, Nationwide Inpatient Sample	No—Study broadly assessed characteristics	Race, sex	Increased risk: female sex (aOR 1.29, 95% CI 1.24–1.35) Decreased risk: Hispanic ethnicity (aOR 0.75, 95% CI 0.7–0.79) No change: Black race (aOR 0.97, 95% CI 0.91–1.04)
Tilton (2019) **No**	Adults admitted to the hospital receiving systemic antibiotics N = 200	North Carolina	HO-CDI	Retrospective case-control, 2015–2017, Wake Forrest University data set	No—Study broadly assessed characteristics	Age	Increased risk: Age > 70 (aOR 1.89, 95% CI 1.05–3.43)
Vader (2021) **Yes**	Adults from Philadelphia county admitted to hospital N = 494	Pennsylvania	HO-CDI	Retrospective case-control, 2014–2018, Drexel University database	Yes	Race, insurance status, census tract	Increased risk: Medicare insurance (aOR 2.04, 95% CI 1.31–3.2) No difference: Black race (aOR 1.04, 95% CI 0.62–1.73) Hispanic ethnicity (aOR 0.71, 95% CI 0.25–2.01) Medicaid insurance (ref private) (aOR 1.76, 95% 0.98–3.15) Census tract (no effect estimate available)
Other Outcomes							
Bakullari (2014) **Yes**	Adults hospitalized with acute cardiovascular disease, pneumonia, or major surgery N = 79,019	Across United States	Pooled HAI event (HO-CDI, CLABSI, CAUTI, HO-MRSA, HO-PNA, SSI)	Retrospective cohort, 2009–2011, Medicare Patient Safety Monitoring System	Yes	Race, ethnicity	Increased risk: Hispanic ethnicity (aOR1.3 95% CI 1.15–1.53), Asian race (aOR 1.4 95% CI 1.07–1.75) No change: Black Race (aOR 1.1 95%CI 0.99–1.23), Native Hawaiian/ Pacific Islander (aOR 0.7, 95% CI 0.4–1.12)
Burke (2009) **No**	Children admitted with S. Aureus bacteremia N = 151	California	HO-MRSA bacteremia	Retrospective cohort, 2001–2006, Stanford University data set	No—Study broadly assessed characteristics	Race, age	Decreased risk: Hispanic ethnicity (aOR 0.16, 95% CI 0.03–0.96) Newborn (aOR 0.21, 95% CI 0.05–0.82) Increased risk: age 6–12 months (ref > 1 year) (aOR 2.4, 95% CI 1.04–5.58)
Gualandi (2018) **No**	Adults and children admitted with MRSA infection N = 9,019	Across United States	HO-MRSA	Retrospective cohort, 2005–2014, Emerging Infections Program data set	Yes	Race, sex	Increased risk: Black race (RR 3.2, 95% CI 2.35–4.35) Decreased risk: Female sex (RR 0.71, 95% CI 0.53–0.97)
Jeon (2014) **No**	Hospitalized Adults N = 60,994	New York	HO-BSI	Retrospective cohort, 2006–2008, Columbia University data set	Yes	Race, age, sex, income, insurance	Increased risk: Black race (HR 1.31, 95% CI 1.02–1.69) Male sex (HR 2.8, 95% CI 1.59–4.94) Medicaid insurance (ref private) (HR 1.34, 95% CI 1.02–1.75) No change: Hispanic ethnicity (HR 1.19, 95% CI 0.92–1.54) Neighborhood median income (HR 1.1, 95% CI 0.77–1.57),
Liu (2020) **No**	Very low birth weight infants N = 20,692	California	HO-BSI	Retrospective Cohort, 2011–2015, California Perinatal Quality Care Collaborative	No—Study broadly assessed characteristics	race	Increased risk: Hispanic ethnicity (aOR 1.23, 95% CI 1.06–1.41) No change: Black race (aOR 1.12, 95% CI 0.93–1.35) Asian/pacific islander race (aOR 1.11, 95% CI 0.9–1.35) “Other” race (aOR 1.09, 95% CI 0.78–1.52)
Milstone (2011) **No**	Children admitted to the pediatric ICU N = 3,620	Maryland	HO-MRSA	Retrospective cohort, 2007–2010, Johns Hopkins Hospital data set	No—Study broadly assessed characteristics	Race, insurance	Increased risk: Black race (*P* < .01, no effect estimate given) Medicaid insurance (ref private) (*P* < .03, no effect estimate given)
Ricciardi (2008) **No**	Adults undergoing appendectomy, gastric bypass or fundoplication N = 88,545	Across United States	HO-PNA Mortality	Retrospective cohort, 2004, Nationwide Inpatient Sample	Yes	Race, age, sex, income, rurality, insurance status	**Mortality data:** Increased risk: Black race (aOR 2.01, 95% CI 1.1–3.69), male sex (aOR 2.1, 95% CI 1.47–2.99) increasing age (by decade) (aOR 1.58, 95% CI 1.4–1.79) Medicare and Medicaid insurance (rel to private) (aOR 3.91, 95% CI 2.4–6.36) No change: Hispanic ethnicity (aOR 1.33, 95% CI 0.67–2.63) Asian race (aOR 1.54, 95% CI 0.46–5.2) income <$36,000 per year (aOR 1.58, 95% CI 0.89–2.8) rural (ref urban) (aOR 0.68, 95% CI 0.31–1.49) **HO-PNA:** Increased risk: Black race (aOR 1.5, 95% CI 1.14–1.97)
Zarzaur (2013) **No**	Adults admitted to trauma service requiring intubation for >2 days N = 5,195	Tennessee	VAP	Retrospective cohort, 1996–2010, University of Tennessee Health Science Center	Yes	Race, sex, income, geography	Decreased risk: female sex (aOR 0.91, 95% CI 0.88–0.94), non-white race (aOR 0.94, 95% CI 0.91–0.97) No change: Median income of home zip code (No effect estimate given) Variable change: Home zip code (No effect estimate given)

*Note:* White race is the reference group for all racial comparisons. Private insurance is the reference group for all insurance comparisons unless otherwise specified.

### Surgical site infection

SSI was the most commonly studied HAI (*n* = 23), representing over half of the included articles. Most studies evaluated differences in patient outcomes for a single category of procedures (e.g., orthopedic surgery); only three studies evaluated surgical patients more broadly.^7,14,18^ The most frequently studied surgical categories were orthopedic surgeries (*n* = 8), bowel surgeries (*n* = 5), and gynecologic surgeries (*n* = 3). There was a high degree of heterogeneity across studies regarding patient inclusion/exclusion criteria, definitions of the SSI outcome, and strategies for dealing with incomplete racial and ethnicity data. Despite this heterogeneity in approach, we determined all included studies were methodologically sound on the basis of our risk of bias assessments. Sufficient data were available to perform meta-analysis for race, ethnicity, sex, and type of medical insurance.

**Figure 2.**
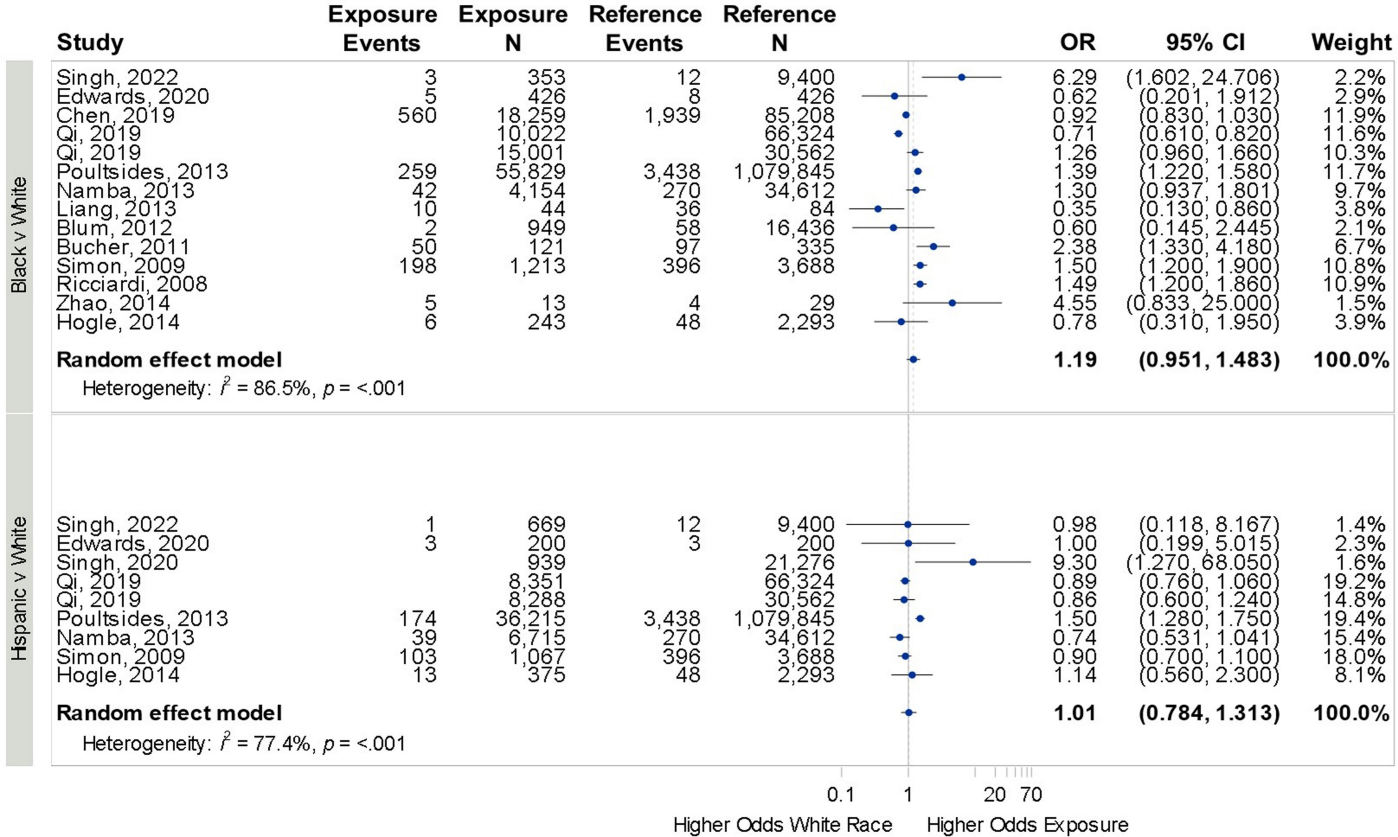
Figure 2 long description. Forrest plot for odds of surgical site infection across race and ethnicity. Individual study details are available in Table 1. Surgeries were diverse including total knee, hip, or shoulder arthroplasty, hysterectomy, bariatric surgery, colon surgery, genital reconstruction, and other general surgeries.

Twenty studies compared SSI rates between Black and White patients;^7,14–16,18–27,29–33,35^ 14 were included in the statistical analysis. Eleven studies compared SSI among Hispanic and White patients,^7,14,20,21,24–27,29–31^ nine of which were included in the statistical analysis. Six studies compared SSI among Black and Hispanic patients. No difference was seen in SSI across any of the racial and ethnic comparisons: Black vs White: OR 1.19 (95% CI 0.95–1.48, *I* ^2^ = 86.5%), Hispanic vs White: OR 1.01 (95% CI 0.78–1.31, *I* ^2^ = 77.4%), (Figure 2). Eight studies compared SSI rates between male and female patients,^14,22–26,28,29^ seven of which were included in the statistical analysis. Female sex was associated with a lower SSI rate : OR 0.74 (95% CI 0.54–0.97, *I* ^2^ = 90.6%, S7). Five studies compared outcomes in patients with public vs private insurance,^14,17,27,30,34^ three of which were included in the statistical analysis. Medicare insurance was associated with a higher SSI incidence: OR 1.28 (95% CI 1.16–1.41, *I* ^2^ = 100%, S8).

The low study number and significant methodological heterogeneity precluded meta-analysis of other SDOH. Specifically, studies were inconsistent in their definitions of age groups, precluding a formal meta-analysis of the risk of age on SSI, and individual studies showed mixed results for the interaction between age and SSI. One study evaluated patient income as a risk factor for SSI, demonstrating low income to be a risk factor.^27^ One study found an association between low health literacy (defined by a score <12 on the Brief Health Literacy Screen) and SSI.^32^ Three studies suggested an increased SSI risk in patients with obesity, though insufficient information was provided in these studies to perform meta-analysis.^22,24,25^

### Clostridioides difficile

**Figure 3.**
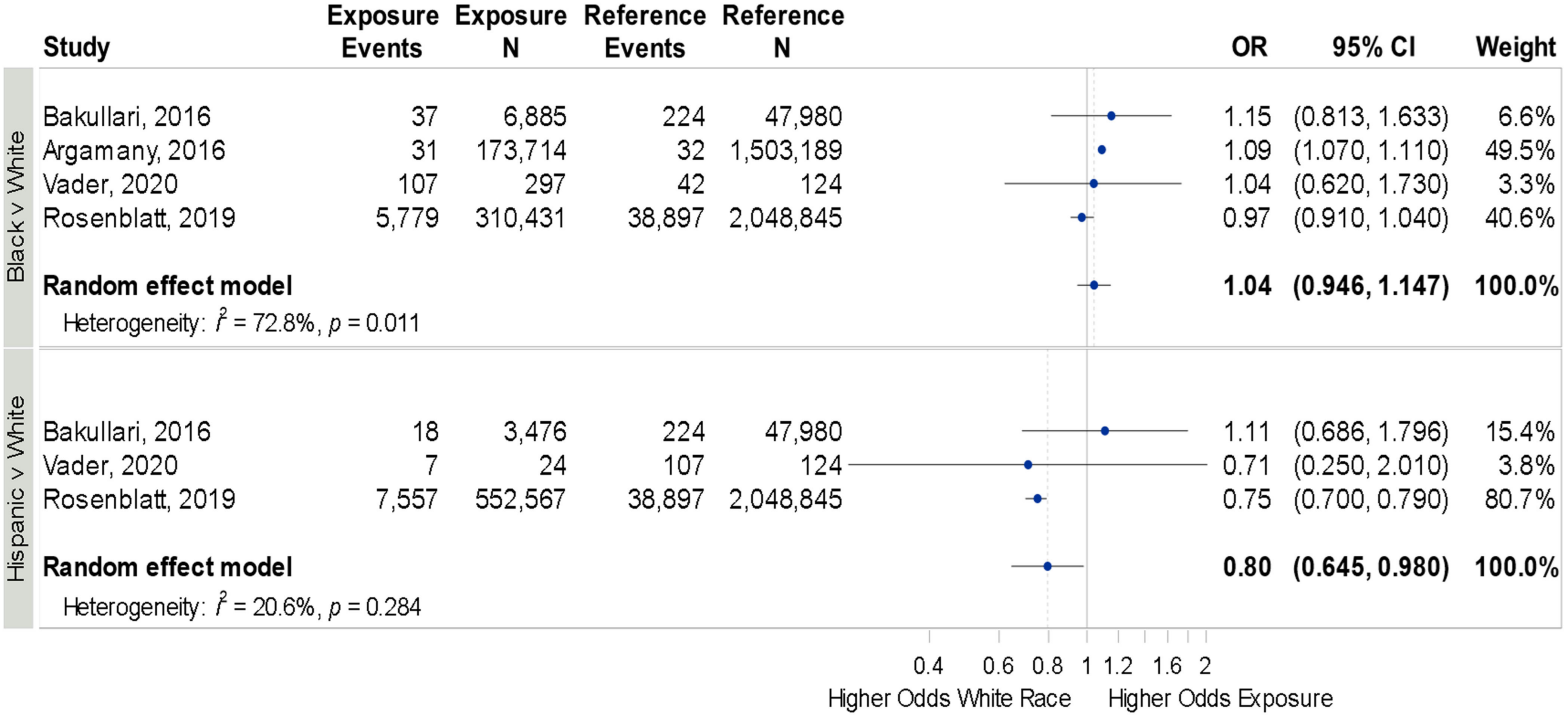
Figure 3 long description. Forrest plot for odds of hospital-onset *Clostridioides difficile* infection across race and ethnicity. Individual study details are available in Table 1.

Of the 6 studies examining HO-CDI, 3 evaluated all hospitalized patients^7,40,44^ while the other 3 evaluated distinct populations of inpatients (patients receiving systemic antibiotics,^43^ patients with advanced cirrhosis,^42^ or patients undergoing major vascular surgery^41^), again resulting in significant heterogeneity across the included studies. Four studies evaluated race, ethnicity and HO-CDI,^7,40,42,44^ 3 of which were included in the meta-analysis. No difference in HO-CDI was seen between Black and White patients (OR 1.04, 95% CI 0.93–1.14, *I* ^2^ = 72.8%), though Hispanic patients had a lower HO-CDI risk compared to White patients (OR 0.80, 95% CI 0.64–0.98, *I* ^2^ = 20.6%, Figure 3).

Of studies not included in the meta-analysis, 2 studies evaluated sex, only 1 of which showed female sex to be a risk factor for HO-CDI.^41,42^ One study evaluated patient census tract and demonstrated no change in risk of HO-CDI based on residential address.^44^ One study showed a significant association between HO-CDI and age > 70.^43^ Another single study showed a decreased HO-CDI risk when hospitalized in a rural setting.^41^ Two studies reported an increased risk of HO-CDI associated with Medicare health insurance compared to private insurance (Table 1).^41,44^

### Central line associated bloodstream infections and hospital onset bloodstream infections

**Figure 4.**
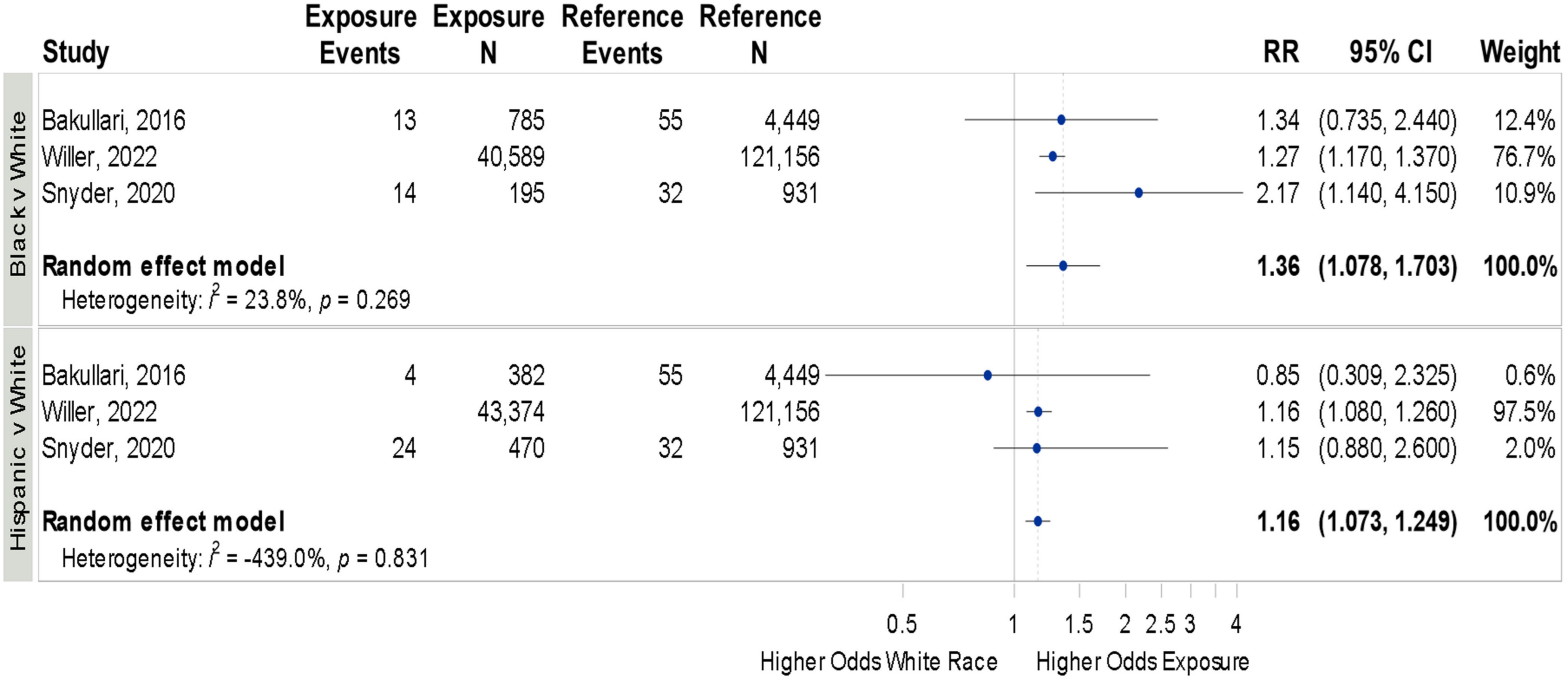
Figure 4 long description. Forrest plot for odds of central line associated bloodstream infections across race and ethnicity. Individual study details are available in Table 1.

Five studies evaluated CLABSI with substantial heterogeneity in patient populations and clinical context; one exclusively included neonates,^38^ one evaluated all pediatric patients,^39^ and three included adults on single units or with a specific diagnosis.^7,36,37^ Four studies evaluated race and CLABSIs,^7,37–39^ three of which (two pediatric and one adult) were included in the meta-analysis. Both Black and Hispanic patients had higher CLABSI rates compared to White patients: Black vs White OR 1.36 (95% CI 1.07–1.70,*I* ^2^ = 23.8%), Hispanic vs White OR 1.16 (95% CI 1.07–1.24, *I* ^2^ = 100%; Figure 4).

Of studies not included in meta-analyses, one showed female sex was associated with a lower CLABSI incidence (Table 1).^37^ In one pediatric study examining HO-BSI, age less than 1 year (excluding neonates hospitalized at birth) was associated with higher HO-BSI rates;^45^ younger age (as a continuous linear variable) was associated with lower CLABSI risk in an adult study.^37^ One study of HO-BSI found no association with patient income.^46^ Two studies found that public insurance was associated with a higher CLABSI or HO-BSI incidence.^36,46^ One study explored patient localities and found that patients from suburban areas (ref: central metropolitan, population >1 million) and location in the southern US (ref: Northeast US) had higher CLABSI incidences.^38^

### Other HAIs and exposures

No other meta-analyses could be performed from the remaining extracted studies and data. Two studies examined risk factors for ventilator-associated pneumonia (VAP) or hospital-onset pneumonia, with one suggesting an increased risk in patients of Black race and the other suggesting a decreased risk for “non-White” race.^49,50^ Median income of home zip code was not associated with a change in VAP rates, while female sex was associated with a lower incidence of VAP.^50^

Three studies examined various hospital-onset MRSA infections (e.g., SSI, skin infection, and BSI).^8,14,48^ All of these identified Black race as a risk factor, but were inconsistent on the effects observed for Hispanic and Asian ethnic and racial groups. Two manuscripts suggested female sex was associated with a lower MRSA incidence.^8,14^ A single study suggested public health insurance was associated with an increased risk of MRSA infections relative to private insurance.^48^

## Discussion

To our knowledge, this is the first systematic review and meta-analysis to evaluate the association of race and ethnicity with reportable HAIs such as SSI, HO-CDI, and CLABSI. We found that 1) methods used to evaluate race, ethnicity, SDOH, and HAI outcomes were highly heterogeneous and 2) the presence and directionality of differences between racial and ethnic groups varied based on the underlying disease process. During the selection process, we also reviewed the papers for additional SDOH measures. Public health insurance remained a persistent risk factor compared to private health insurance across almost all types of HAI; otherwise, most SDOH evaluated in our study varied by disease process. Most significantly, however, our study highlighted the paucity of data looking at the influence of most SDOH on HAI disparities, particularly in pediatric populations.

Although studies identifying racial and ethnic disparities in HAIs are increasing, this area of research is relatively new.^51^ Few studies have risen to the level of systematic reviews or meta-analyses. In a previous study, our group identified 2 other systematic reviews that evaluated the associations between race, ethnicity, and SDOH with SSIs.^5^ Both of these studies looked exclusively at neurosurgical patients; one showed an association between SSI and “non-white” race, and the other showed increased SSI risk in obese patients.^52,53^ Our current review includes data from a larger array of surgical procedures and a more diverse set of HAIs. The more expansive scope of this review is novel in emphasizing which combinations of SDOH domains and HAIs need further investigation.

Our analysis identified significant differences across racial and ethnic groups in CLABSI and HO-CDI but not amongst SSIs. Insufficient data existed to analyze other HAIs. It is unclear if these differences represent the unique pathophysiology and risk factors of these infections or are simply related to the lower number of studies in the CLABSI and HO-CDI analyses (fourteen SSI vs four HO-CDI and three CLABSI). Our search strategy focused on race and ethnicity with only secondary review of other SDOH, limiting our ability to make this determination. It is notable that beyond race, ethnicity, and sex, demographics required for most research, the only SDOH studied consistently enough to allow for meta-analysis was insurance status.

In preparing this manuscript, the authorship group is aware of the problematic nature of racial categories in clinical research. In American society, racial categorizations are primarily aggregate indicators of the degree of structural racism (including adverse SDOH profiles) that individuals have been exposed to over the course of their lives in our society. We fully acknowledge that racial categories are a social construct and have essentially no basis in biology, and our findings based on this reality do not imply any genetic associations with the pathophysiology of the observed disease states. However, in retrospect, we have determined that the study of racial associations with HAIs provide a crude yet useful insight into the effect of structural racism and racialization on the health outcomes of individuals stratified by race in the US. Given the limitations of our retrospective review (and the race-based variable collected in prior reports) further study is needed to better identify the precise drivers of disparate HAI outcomes among persons of different racial strata in the American context. The differences we observed by race in this meta-analysis provide the critical premise to justify further study along this line of investigation.^54–57^

Few studies that examine racial and ethnic disparities consider the SDOH for which race and ethnicity may be a marker. More research is needed to disentangle the interdependent relationships of SDOH, race, and ethnicity before we can fully understand their independent effects. Systematic reviews with a narrower scope may provide further insight to these relationships. We initially attempted to use Boolean operators in our search that would look for any analysis of race, ethnicity or SDOH. This approach resulted in millions of articles and made for an untenable approach. We subsequently limited our search to race and ethnicity. By asking more targeted questions with specific SDOH variables and stricter inclusion criteria, more robust meta-analysis may be possible.

Two additional striking features of our review were the lack of prospective data and the dependence on readily available local or national databases such as the National Surgical Quality Improvement Program (NSQIP) or the National Inpatient Sample for outcome determination. Finding ways to incentivize the routine and accurate documentation of race, ethnicity, and SDOH will be essential to better understand health disparities in HAIs.

Our study has the following limitations. First, HAI outcomes largely used ICD codes for diagnosis or data available from large data sets as opposed to formal NHSN definitions for HAIs of interest. Additionally, included manuscripts had variable approaches for categorizing racial and ethnic groups, analyses of multiracial individuals, and accounting for patients with missing race or ethnicity data. For example, some studies considered Hispanic as a race mutually exclusive to other categories, and others considered it an ethnicity to be combined with other racial groups. Furthermore, some manuscripts focused on specific age ranges or conditions limiting generalizability to larger populations. Together these factors resulted in a very high level of heterogeneity across our included studies with most *I* ^2^ values >75%. As a result, we recommend strong caution with interpretation of statistical inference from our analyses. Second, many studies identified by our systematic review did not include sufficient primary data to allow for inclusion in our statistical analysis. In addition, as this review comes from the published literature, and many of the included studies did not prespecify a health equity focus, it is possible that studies demonstrating a racial or ethnic difference are overrepresented secondary to publication bias. Third, our search may not have identified all published studies that were applicable to our study objectives. For example, an additional search by the authors using their own terms was able to identify additional potentially relevant articles not originally identified.^58–63^ Despite these limitations, we believe this review is a valuable summary of the current landscape.

## Conclusion

The study findings highlight potential racial and ethnic disparities in CLABSI and HO-CDI rates, but further work is needed to standardize how race and ethnicity are defined and collected across all electronic health record systems. Other SDOH, including healthcare access and support, community context, and economic stability are likely important drivers but, similarly, more research is needed to better elucidate these relationships. To facilitate robust health equity research, HAI surveillance, and prevention efforts, we need standardized definitions, and validated questions to report race, ethnicity, and social risk factors.

## Supporting information

10.1017/ice.2026.10461.sm001 Perez et al. supplementary material Perez et al. supplementary material

## Data Availability

Data used in this study are readily available in the referenced publications.

## Supplementary material

The supplementary material for this article can be found at https://doi.org/10.1017/ice.2026.10461.

## Figure 1 Long description

The flowchart begins with the identification stage, where studies from databases and registers total 9664, and references from other sources are zero. It then shows the removal of 4578 references due to duplicates and other reasons. The screening stage involves 5086 studies, with 4883 studies excluded, leaving 196 studies sought for retrieval. All 196 studies are retrieved and assessed for eligibility, with 157 studies excluded for various reasons such as wrong setting, wrong outcomes, and wrong comparator. Finally, 39 studies are included in the review.

Navigate back to Figure 1.

## Figure 2 Long description

A horizontal dot plot compares the odds of surgical site infection across different races and ethnicities. The plot is divided into two sections: Black vs. White and Hispanic vs. White. Each section lists multiple studies with their respective data points. The x-axis represents the odds ratio (OR) ranging from 0.1 to 20, while the y-axis lists the studies involved. Each dot represents the OR for a specific study, with horizontal lines indicating the 95 percent confidence interval (CI). The weight percentage of each study is also shown. The Black vs. White section includes studies from 2008 to 2022, with a random effect model OR of 1.19 and a heterogeneity of 86.5 percent. The Hispanic vs. White section includes studies from 2009 to 2022, with a random effect model OR of 1.01 and a heterogeneity of 77.4 percent. The plot highlights variations in infection odds across different racial and ethnic groups, with some studies showing higher odds for certain groups. All values are approximated.

Navigate back to Figure 2.

## Figure 3 Long description

The table presents a comparison of the odds of hospital-onset Clostridioides difficile infection across different races and ethnicities. It includes data from multiple studies, with columns for exposure events, exposure sample size, reference events, reference sample size, odds ratio (OR), confidence interval (CI), and weight. The studies compare Black versus White and Hispanic versus White populations. For Black versus White, the studies are Bakullari, 2016; Argaman, 2016; Vader, 2020; and Rosenblatt, 2019. For Hispanic versus White, the studies are Bakullari, 2016; Vader, 2020; and Rosenblatt, 2019. The random effect models show an overall OR of 1.04 for Black versus White and 0.80 for Hispanic versus White. The table highlights the heterogeneity and p-values for each model.

Navigate back to Figure 3.

## Figure 4 Long description

The bar graph compares the odds of central line associated bloodstream infections across different races and ethnicities. It features horizontal bars representing the relative risk (RR) with 95% confidence intervals (CI) for each study. The x-axis ranges from 0.5 to 4, indicating the higher odds for white race versus exposure. The y-axis lists the studies by authors and years: Bakulari, 2016; Willer, 2022; Snyder, 2020. The graph is divided into two sections: Black vs. White and Hispanic vs. White. Each section includes data from the three studies, with the random effect model summarized at the bottom. The bars show the RR values and their CI, with weights assigned to each study. The color scheme uses blue for the data points and lines. The graph highlights the heterogeneity and p-values for the random effect models. All values are approximated.

Navigate back to Figure 4.

## Table 1 Long description

A table with 39 rows and 7 columns summarizing healthcare-associated infection studies. The columns include Study ID, Design, Population, Infection Type, Exposures, Results, and Notes. The table details various study designs such as retrospective cohort, prospective cohort, and retrospective case-control. It lists different infection types like SSI, CDI, CLABSI, and other HAIs. The exposures studied include race, ethnicity, age, sex, and insurance status. The results and notes provide specific findings from each study.

Navigate back to Table 1.
