# Supplementary material for: Racial disparities in healthcare-associated infections: a systematic review and meta-analysis
Source: Infect Control Hosp Epidemiol. 2026 Apr 24;47(6):553–67. doi: 10.1017/ice.2026.10461 (PMC13216807; doi:10.1017/ice.2026.10461)
Supplement: Perez et al. supplementary material [file S0899823X26104619sup001.docx]

**Supplement**

**S1. Search Strategies**

Steph Hendren, MLIS; Duke University Medical Center Library, Duke University School of Medicine

Date of completed search: January 12, 2023

**Database: Medline (OVID)**

| 1  Race/ethnicity | exp Minority Groups/ OR exp Ethnicity/ OR exp Minority Health/ OR exp racial groups/ OR exp blacks/ OR exp african americans/ OR exp american native continental ancestry group/ OR exp indians, central american/ OR exp indians, north american/ OR exp indians, south american/ OR exp inuits/ OR exp asians/ OR exp asian americans/ OR exp whites/ OR exp "native hawaiian or other pacific islander"/ OR exp ethnicity/ OR exp "hispanic or latino"/ OR exp mexican americans/ OR exp "health disparity, minority and vulnerable populations"/ or exp amish/ or exp arabs/ or exp asian americans/ or exp indigenous peoples/ or exp jews/ or exp roma/ or exp vulnerable populations/ OR (race OR races OR racial OR racially OR ethnicity OR ethnicities OR ethnic OR Hispanic OR Mexican OR Hispanics OR Mexicans OR Cuban OR Cubans OR Latin OR Latina OR Latinas OR Latino OR Latinos OR Latinx OR Latine OR "Latino/a" OR "Latino(a)" OR "Latino/a/x" OR "Latino(a/x)" OR "Latin American" OR "Latin-American" OR "Latin Americans" OR "Latin-Americans" OR "Spanish speaking" OR "Spanish-speaking" OR "Spanish speakers" OR "Spanish-speakers" OR (Spanish AND (speakers OR speaker OR speaking OR language)) OR "Puerto Rico" OR "Puerto Rican" OR "Puerto Ricans" OR "African-American" OR "African American" OR "African-Americans" OR "African Americans" OR Black OR Blacks OR racial OR race OR racism OR racist OR racists OR colorism OR shadeism OR ethnic OR ethnicity OR ethnicities OR minority OR minorities OR indigenous OR ((Native OR natives OR indian OR Indians) AND (American OR Americans OR Alaskan OR Alaskans OR Alaska OR Hawaiian OR Hawaiians)) OR "Pacific Islander" OR "Pacific Islanders" OR Arabs OR Arab OR Arabic OR Palestinian OR Palestinians OR Bedouin OR Bedouins OR Amish OR Mennonite OR Mennonites OR Jews OR Jewish OR Jew OR Roma OR Romany OR Gypsy OR Gypsies OR Romani OR Gipsy OR Gipsies OR Indigenous OR "First Nation" OR "first nations" OR Native OR Oceanic OR Burmese OR Cambodian OR Vietnamese OR Japanese OR Korean OR Koreans OR Mongoloid OR Asiatic OR Thai OR Asian OR Asians OR Chinese OR Filipino OR Filipinos OR Indian OR Indians OR Hmong OR Pakistani OR Pakistanis OR Iranian OR Iranians OR Iraqi OR Iraqis OR Tibetans OR Tibetan OR Nepalese OR Kurd OR Kurds OR Armenian OR Armenians OR Punjabi OR Punjabis OR Kazakhs OR Kazakh OR Uyghurs OR Uyghur OR "Sri Lankan" OR Taiwanese OR BIPOC OR "people of color" OR "person of color" OR "persons of color").ti,ab. | 1686449 |
| --- | --- | --- |
| 2  HAIs (CLABSIs, CAUTIs, C.difficile, SSIs, HO-BSI, MRSA) | (("central line" OR "central venous") adj3 (infection OR infections)).ti,ab. OR ((exp Catheter-Related Infections/ AND ("central line" OR "central venous").ti,ab.)) OR (exp Catheterization, Central Venous/ AND (Infections/ OR (infection OR infections).ti,ab.)) OR ((healthcare OR "health care" OR hospital OR hospitals OR surgical OR surgically) adj3 (infection OR infections OR infected)).ti,ab. OR ((catheters, indwelling/ OR urinary catheters/ OR urinary catheterization/ OR ("urinary cathether" OR "urinary catheters").ti,ab.) AND ((Catheter-Related Infections/ OR Urinary Tract Infections/) OR (infection OR infections).ti,ab.)) OR (Clostridiodes difficile/ OR Clostridium Infections/ OR ("c diff" OR "clostridium difficile" OR "clostridioides difficile").ti,ab.) OR ("hospital onset bloodstream infection" OR "hospital-onset bloodstream infection" OR "hospital onset bloodstream infections" OR "hospital-onset bloodstream infections" OR "mrsa bacteremia" OR "methicillim resistant" OR methicillin-resistant OR ("Staphylococcus aureus" AND resistant)).ti,ab. OR (CLABSI OR CLABSIs OR HAI OR HAIs OR CAUTI OR CAUTIs OR HOBSI OR HOBSIs OR HO-BSI OR HO-BSIs OR SSI OR SSIs).ti,ab. | 131811 |
| 3  Inpatient | exp Inpatients/ OR exp hospitalization/ OR exp hospital units/ OR hospitals/ OR (inpatient* OR in-patient* OR hospital*).ti,ab. | 3538466 |
| 4 | 1 AND 2 AND 3 | 3065 |
| 5 | Limit 4 to da=20080101-20231231 | 2560 |

**Database: Embase (Elsevier)**

| 1  Race/ethnicity | 'health disparity'/exp OR 'minority group'/exp OR 'ethnic group'/exp OR 'race'/exp OR 'race difference'/exp OR 'ethnicity'/exp OR 'ethnic difference'/exp OR 'ancestry group'/exp OR 'Mexican American'/exp OR 'African American'/exp OR 'Black person'/exp OR 'Asian continental ancestry group'/exp OR 'Asian American'/exp OR 'American Indian'/exp OR 'Asian American'/exp OR 'Arab'/exp OR 'Amish'/exp OR 'Jew'/exp OR 'Romani (people)'/exp OR 'indigenous people'/exp OR 'structural racism'/exp OR 'American Indian'/exp OR 'Oceanic ancestry group'/exp OR 'Native Hawaiian'/exp OR (Hispanic OR Mexican OR Hispanics OR Mexicans OR Cuban OR Cubans OR Latin OR Latina OR Latinas OR Latino OR Latinos OR Latinx OR Latine OR latines OR 'Latino/a' OR 'Latino(a)' OR 'Latino/a/x' OR 'Latino(a/x)' OR 'Latin American' OR 'Latin-American' OR 'Latin Americans' OR 'Latin-Americans' OR 'Spanish speaking' OR 'Spanish-speaking' OR 'Spanish speakers' OR 'Spanish-speakers' OR (Spanish AND (speakers OR speaker OR speaking OR language)) OR 'Puerto Rico' OR 'Puerto Rican' OR 'Puerto Ricans' OR 'African-American' OR 'African American' OR 'African-Americans' OR 'African Americans' OR Black OR Blacks OR racial OR race OR ethnic OR ethnicity OR ethnicities OR minority OR minorities OR indigenous OR ((Native OR natives OR indian OR Indians) AND (American OR Americans OR Alaskan OR Alaskans OR Alaska OR Hawaiian OR Hawaiians)) OR 'Pacific Islander' OR 'Pacific Islanders' OR Polynesians OR Polynesian OR Arabs OR Arab OR Arabic OR Palestinian OR Palestinians OR Bedouin OR Bedouins OR Amish OR Mennonite OR Mennonites OR Jews OR Jewish OR Jew OR Roma OR Romany OR Gypsy OR Gypsies OR Romani OR Gipsy OR Gipsies OR Indigenous OR 'First Nation' OR 'first nations' OR Native OR Oceanic OR Burmese OR Cambodian OR Vietnamese OR Japanese OR Korean OR Koreans OR Mongoloid OR Asiatic OR Thai OR Asian OR Asians OR Chinese OR Filipino OR Filipinos OR Indian OR Indians OR Hmong OR Pakistani OR Pakistanis OR Iranian OR Iranians OR Iraqi OR Iraqis OR Tibetans OR Tibetan OR Nepalese OR Kurd OR Kurds OR Armenian OR Armenians OR Punjabi OR Punjabis OR Kazakhs OR Kazakh OR Uyghurs OR Uyghur OR 'Sri Lankan' OR Taiwanese OR BIPOC OR 'person of color' OR 'persons of color' OR 'people of color'):ti,ab | 2358386 |
| --- | --- | --- |
| 2  HAIs (CLABSIs, CAUTIs, C.difficile, SSIs, HO-BSI, MRSA) | 'central line infection'/exp OR (('central line' OR 'central venous') adj3 (infection OR infections)):ti,ab OR (('catheter infection'/exp AND ('central line' OR 'central venous'):ti,ab)) OR ('central venous catheter'/exp OR 'central venous catheterization'/exp AND ('infection'/exp OR (infection OR infections):ti,ab)) OR ((healthcare OR 'health care' OR hospital OR hospitals OR surgical OR surgically) adj3 (infection OR infections OR infected)):ti,ab OR (('indwelling catheter'/exp OR 'urinary catheter'/exp OR 'bladder catheterization'/exp OR ('urinary cathether' OR 'urinary catheters'):ti,ab) AND (('catheter infection'/exp OR 'urinary tract infection'/exp) OR (infection OR infections):ti,ab)) OR ('Clostridioides'/exp OR 'Clostridium infection'/exp OR ('c diff' OR 'clostridium difficile' OR 'clostridioides difficile'):ti,ab) OR ('hospital onset bloodstream infection' OR 'hospital-onset bloodstream infection' OR 'hospital onset bloodstream infections' OR 'hospital-onset bloodstream infections' OR 'mrsa bacteremia' OR 'methicillim resistant' OR methicillin-resistant OR ('Staphylococcus aureus' AND resistant)):ti,ab OR (CLABSI OR CLABSIs OR HAI OR HAIs OR CAUTI OR CAUTIs OR HOBSI OR HOBSIs OR HO-BSI OR HO-BSIs OR SSI OR SSIs):ti,ab | 180834 |
| 3  Inpatient | 'hospital patient'/exp OR 'hospitalization'/exp OR 'hospital'/exp OR (inpatient* OR in-patient* OR hospital*):ti,ab | 5958891 |
| 4 | 1 AND 2 AND 3 | 4951 |
| 5 | 4 NOT ('editorial'/exp OR [editorial]/lim OR 'letter'/exp OR [letter]/lim OR 'note'/exp OR [note]/lim OR [conference abstract]/lim OR 'conference abstract'/exp OR 'conference abstract'/it) | 3083 |

Database: CINAHL Complete (EBSCOhost)

| 1  Race/ethnicity | MH "Minority Groups" OR MH "Racial Equality" OR MH "Ethnic Groups+" OR MH "Amish" OR MH "Arabs" OR MH "Asians+" OR MH "Cambodians" OR MH "Chinese" OR MH "Filipinos" OR MH "Hmong" OR MH "Japanese" OR MH "Koreans" OR MH "Laotians" OR MH "Thais" OR MH "Vietnamese" OR MH "Black Persons+" OR MH "African Americans" OR MH "Roma" OR MH "Hispanic Americans+" OR MH "Mexican Americans" OR MH "Indigenous Peoples+" OR MH "Aboriginal Canadians+" OR MH "Arctic Peoples+" OR MH "First Nations of Australia+" OR MH "Maori" OR MH "Native Americans+" OR MH "First Nations of Canada" OR MH "Inuit" OR MH "Aboriginal Australians" OR MH "Torres Strait Islanders" OR MH "Alaska Natives" OR MH "Jews" OR MH "Kurds" OR TI (race OR races OR racial OR racially OR ethnicity OR ethnicities OR ethnic OR Hispanic OR Mexican OR Hispanics OR Mexicans OR Cuban OR Cubans OR Latin OR Latina OR Latinas OR Latino OR Latinos OR Latinx OR Latine OR "Latino/a" OR "Latino(a)" OR "Latino/a/x" OR "Latino(a/x)" OR "Latin American" OR "Latin-American" OR "Latin Americans" OR "Latin-Americans" OR "Spanish speaking" OR "Spanish-speaking" OR "Spanish speakers" OR "Spanish-speakers" OR (Spanish AND (speakers OR speaker OR speaking OR language)) OR "Puerto Rico" OR "Puerto Rican" OR "Puerto Ricans" OR "African-American" OR "African American" OR "African-Americans" OR "African Americans" OR Black OR Blacks OR racial OR race OR racism OR racist OR racists OR colorism OR shadeism OR ethnic OR ethnicity OR ethnicities OR minority OR minorities OR indigenous OR ((Native OR natives OR indian OR Indians) AND (American OR Americans OR Alaskan OR Alaskans OR Alaska OR Hawaiian OR Hawaiians)) OR "Pacific Islander" OR "Pacific Islanders" OR Arabs OR Arab OR Arabic OR Palestinian OR Palestinians OR Bedouin OR Bedouins OR Amish OR Mennonite OR Mennonites OR Jews OR Jewish OR Jew OR Roma OR Romany OR Gypsy OR Gypsies OR Romani OR Gipsy OR Gipsies OR Indigenous OR "First Nation" OR "first nations" OR Native OR Oceanic OR Burmese OR Cambodian OR Vietnamese OR Japanese OR Korean OR Koreans OR Mongoloid OR Asiatic OR Thai OR Asian OR Asians OR Chinese OR Filipino OR Filipinos OR Indian OR Indians OR Hmong OR Pakistani OR Pakistanis OR Iranian OR Iranians OR Iraqi OR Iraqis OR Tibetans OR Tibetan OR Nepalese OR Kurd OR Kurds OR Armenian OR Armenians OR Punjabi OR Punjabis OR Kazakhs OR Kazakh OR Uyghurs OR Uyghur OR "Sri Lankan" OR Taiwanese OR BIPOC OR "people of color" OR "person of color" OR "persons of color") OR AB (race OR races OR racial OR racially OR ethnicity OR ethnicities OR ethnic OR Hispanic OR Mexican OR Hispanics OR Mexicans OR Cuban OR Cubans OR Latin OR Latina OR Latinas OR Latino OR Latinos OR Latinx OR Latine OR "Latino/a" OR "Latino(a)" OR "Latino/a/x" OR "Latino(a/x)" OR "Latin American" OR "Latin-American" OR "Latin Americans" OR "Latin-Americans" OR "Spanish speaking" OR "Spanish-speaking" OR "Spanish speakers" OR "Spanish-speakers" OR (Spanish AND (speakers OR speaker OR speaking OR language)) OR "Puerto Rico" OR "Puerto Rican" OR "Puerto Ricans" OR "African-American" OR "African American" OR "African-Americans" OR "African Americans" OR Black OR Blacks OR racial OR race OR racism OR racist OR racists OR colorism OR shadeism OR ethnic OR ethnicity OR ethnicities OR minority OR minorities OR indigenous OR ((Native OR natives OR indian OR Indians) AND (American OR Americans OR Alaskan OR Alaskans OR Alaska OR Hawaiian OR Hawaiians)) OR "Pacific Islander" OR "Pacific Islanders" OR Arabs OR Arab OR Arabic OR Palestinian OR Palestinians OR Bedouin OR Bedouins OR Amish OR Mennonite OR Mennonites OR Jews OR Jewish OR Jew OR Roma OR Romany OR Gypsy OR Gypsies OR Romani OR Gipsy OR Gipsies OR Indigenous OR "First Nation" OR "first nations" OR Native OR Oceanic OR Burmese OR Cambodian OR Vietnamese OR Japanese OR Korean OR Koreans OR Mongoloid OR Asiatic OR Thai OR Asian OR Asians OR Chinese OR Filipino OR Filipinos OR Indian OR Indians OR Hmong OR Pakistani OR Pakistanis OR Iranian OR Iranians OR Iraqi OR Iraqis OR Tibetans OR Tibetan OR Nepalese OR Kurd OR Kurds OR Armenian OR Armenians OR Punjabi OR Punjabis OR Kazakhs OR Kazakh OR Uyghurs OR Uyghur OR "Sri Lankan" OR Taiwanese OR BIPOC OR "people of color" OR "person of color" OR "persons of color") | 474814 |
| --- | --- | --- |
| 2  HAIs (CLABSIs, CAUTIs, C.difficile, SSIs, HO-BSI, MRSA) | TI (("central line" OR "central venous") N3 (infection OR infections)) OR AB (("central line" OR "central venous") N3 (infection OR infections)) OR ((MH "Catheter-Related Infections+" OR MH "Catheter-Related Bloodstream Infections") AND (TI ("central line" OR "central venous") OR AB ("central line" OR "central venous"))) OR (MH "Catheterization, Central Venous+" AND (MH "Infection" OR TI (infection OR infections) OR AB (infection OR infection))) OR TI ((healthcare OR "health care" OR hospital OR hospitals OR surgical OR surgically) N3 (infection OR infections OR infected)) OR AB ((healthcare OR "health care" OR hospital OR hospitals OR surgical OR surgically) N3 (infection OR infections OR infected)) OR ((MH "Catheters, Urinary+" OR MH "Urinary Catheterization+" OR TI ("urinary cathether" OR "urinary catheters") OR AB ("urinary cathether" OR "urinary catheters")) AND (MH "Catheter-Related Infections+" OR MH "Urinary Tract Infections+" OR TI (infection OR infections) OR AB (infection OR infections))) OR MH "Urinary Tract Infections, Catheter-Related" OR MH "Clostridioides Difficile" OR MH "Clostridium Infections+" OR TI ("c diff" OR "clostridium difficile" OR "clostridioides difficile") OR AB ("c diff" OR "clostridium difficile" OR "clostridioides difficile") OR TI ("hospital onset bloodstream infection" OR "hospital-onset bloodstream infection" OR "hospital onset bloodstream infections" OR "hospital-onset bloodstream infections" OR "mrsa bacteremia" OR "methicillim resistant" OR methicillin-resistant OR ("Staphylococcus aureus" AND resistant)) OR AB ("hospital onset bloodstream infection" OR "hospital-onset bloodstream infection" OR "hospital onset bloodstream infections" OR "hospital-onset bloodstream infections" OR "mrsa bacteremia" OR "methicillim resistant" OR methicillin-resistant OR ("Staphylococcus aureus" AND resistant)) OR TI (CLABSI OR CLABSIs OR HAI OR HAIs OR CAUTI OR CAUTIs OR HOBSI OR HOBSIs OR HO-BSI OR HO-BSIs OR SSI OR SSIs) OR AB (CLABSI OR CLABSIs OR HAI OR HAIs OR CAUTI OR CAUTIs OR HOBSI OR HOBSIs OR HO-BSI OR HO-BSIs OR SSI OR SSIs) | 46004 |
| 3  Inpatient | MH "Inpatients" OR MH "Hospitalization+" OR MH "Hospital Units+" OR TI (inpatient* OR in-patient* OR hospital*) OR AB (inpatient* OR in-patient* OR hospital*) | 2429202 |
| 4 | 1 AND 2 AND 3 | 1378 |
| 5 | Filter: 2008 – present | 1208 |

**Database: Scopus (Elsevier)**

| 1  Race/ethnicity | TITLE-ABS(race OR races OR racial OR racially OR ethnicity OR ethnicities OR ethnic OR Hispanic OR Mexican OR Hispanics OR Mexicans OR Cuban OR Cubans OR Latin OR Latina OR Latinas OR Latino OR Latinos OR Latinx OR Latine OR {Latino/a} OR {Latino(a)} OR {Latino/a/x} OR {Latino(a/x)} OR {Latin American} OR {Latin-American} OR {Latin Americans} OR {Latin-Americans} OR {Spanish speaking} OR {Spanish-speaking} OR {Spanish speakers} OR {Spanish-speakers} OR (Spanish AND (speakers OR speaker OR speaking OR language)) OR {Puerto Rico} OR {Puerto Rican} OR {Puerto Ricans} OR {African-American} OR {African American} OR {African-Americans} OR {African Americans} OR Black OR Blacks OR racial OR race OR racism OR racist OR racists OR colorism OR shadeism OR ethnic OR ethnicity OR ethnicities OR minority OR minorities OR indigenous OR ((Native OR natives OR indian OR Indians) AND (American OR Americans OR Alaskan OR Alaskans OR Alaska OR Hawaiian OR Hawaiians)) OR {Pacific Islander} OR {Pacific Islanders} OR Arabs OR Arab OR Arabic OR Palestinian OR Palestinians OR Bedouin OR Bedouins OR Amish OR Mennonite OR Mennonites OR Jews OR Jewish OR Jew OR Roma OR Romany OR Gypsy OR Gypsies OR Romani OR Gipsy OR Gipsies OR Indigenous OR {First Nation} OR {first nations} OR Native OR Oceanic OR Burmese OR Cambodian OR Vietnamese OR Japanese OR Korean OR Koreans OR Mongoloid OR Asiatic OR Thai OR Asian OR Asians OR Chinese OR Filipino OR Filipinos OR Indian OR Indians OR Hmong OR Pakistani OR Pakistanis OR Iranian OR Iranians OR Iraqi OR Iraqis OR Tibetans OR Tibetan OR Nepalese OR Kurd OR Kurds OR Armenian OR Armenians OR Punjabi OR Punjabis OR Kazakhs OR Kazakh OR Uyghurs OR Uyghur OR {Sri Lankan} OR Taiwanese OR BIPOC OR {people of color} OR {person of color} OR {persons of color}) | 4139816 |
| --- | --- | --- |
| 2  HAIs (CLABSIs, CAUTIs, C.difficile, SSIs, HO-BSI, MRSA) | TITLE-ABS((line OR venous OR catheter OR catheters) W/3 (infection OR infections)) OR TITLE-ABS ((healthcare OR care OR hospital OR hospitals OR surgical OR surgically) W/3 (infection OR infections OR infected)) OR TITLE-ABS ({c diff} OR {clostridium difficile} OR {clostridioides difficile}) OR TITLE-ABS({hospital onset bloodstream infection} OR {hospital-onset bloodstream infection} OR {hospital onset bloodstream infections} OR {hospital-onset bloodstream infections} OR {mrsa bacteremia} OR {methicillim resistant} OR methicillin-resistant OR ({Staphylococcus aureus} AND resistant)) OR TITLE-ABS(CLABSI OR CLABSIs OR HAI OR HAIs OR CAUTI OR CAUTIs OR HOBSI OR HOBSIs OR HO-BSI OR HO-BSIs OR SSI OR SSIs) | 182572 |
| 3  Inpatient | TITLE-ABS(inpatient* OR in-patient* OR hospital*) | 3532912 |
| 4 | 1 AND 2 AND 3 | 4335 |
| 5 | Publication filter: Article, Review | 4199 |
| 6 | Date limitation: 2008 - present | 3286 |

The databases used were Medline (Ovid), Embase (Elsevier), CINAHL Complete (EBSCOhost), and Scopus (Elsevier). All searches utilized both keywords and, when available, medical subject headings that were related to the key concepts of the research question. Explosion was used on a few select keywords, and adjacency searches were utilized for some portions of the HAI search block. All keywords were required to appear in either the title or abstract of the database citation.

The search was run on January 12, 2023. While no filters for language or publication type was used, a date filter to only include articles from 2008 onwards was used.

Given the broad scope and high number of hits we did not conduct any additional hand searching. Given the importance of social context we excluded articles published in languages other than English as we were only interested in US studies. We did not conduct a search of abstracts or unpublished studies. No attempts were made to contact any authors. We did not conduct an additional analysis for publication bias such as an egger’s test given the breadth of studies included.

**S2: Criteria Used by Authors During Screening and Bias Assessment**

1. **Screening Protocol**

**HAI Systematic Review SOP**

AIM

To understand the association between race and ethnicity and healthcare associated infections, with further review of SDoH measures

**Inclusion Criteria**

1. Does the title/abstract include description of race OR ethnicity?
2. Does it include an HAI? (HAIs include the terms CLABSIs, CAUTIs, CDI, *Staphylococcus aureus* infection, healthcare associated infections, hospital acquired respiratory viruses, healthcare associated-MDROs or gram negatives, nosocomial infections, or surgical site infections)
3. Does it focus on hospitalized or inpatients
   1. Note: By default, surgical populations count as “inpatients” for our purposes
4. Does it describe potential differences in HAI outcomes relative to race, ethnicity, or one or more SDOH?

**Exclusion criteria**

1. Basic or translational science articles
2. Animal models
3. Non-English language
4. Commentary
5. COVID-19 related manuscripts unless discussion of HA- respiratory virus or COVID
6. Impact on healthcare workers


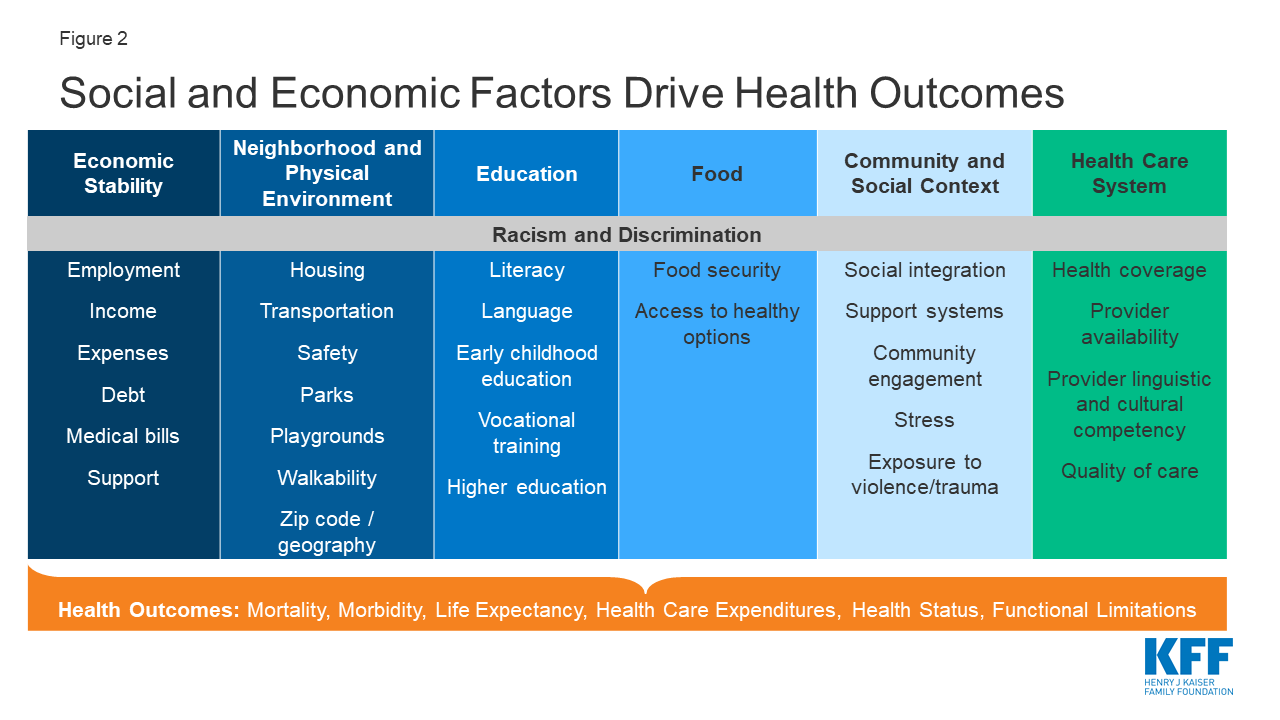


**Other Drivers: Age, Sex, Gender**

General Steps for Review

1. Log into Covidence
2. Read the title and abstract
3. Is there HAI
4. Is there race/ethnicity as a descriptor
5. Is there SDoH (Tag with SDoH or other driver)
6. If choose to include, add tag
7. Notes if desired

**EVALUATION FLOWCHART**

Do not Include

No

Is there a HAI(s) included?

Yes

Is association between SDoH and HAI evaluated?

No

Yes

If Yes, include and mark with applicable Tags (as many as needed)

No

Do not Include

Yes

Yes

Do not Include

No

Is association between race/ethnicity and HAI evaluated?

Is race/ethnicity included?

1. **Full Text Review Protocol**

**HAI Systematic Review SOP – FULL TEXT REVIEW 050823**

**AIM**

To understand the association between race and ethnicity and healthcare associated infections, with further review of SDoH measures *(associations or intersectionality)*

**Reviewer Instructions:**

Read full text of selected article.

Determine if article should be included in analysis. Consider the following inclusion and exclusion criteria:

**Inclusion Criteria**

1. Does the article present data about a specific HAI? To be considered an HAI, the infection onset must begin >=day 3 *of hospitalization*. Specific examples include
   1. CLABSI
   2. HO-BSI
   3. CAUTI
   4. HO-CDI
      1. Note given the labID definition used for C diff studies may not be able to definitively identify this as HAI and we will be accepting of this limitation.
   5. HO-MRSA infection (Bloodstream or otherwise)
   6. VAP
   7. HO-pneumonia
   8. SSI* (*note exception below*)
   9. Other infections that meet onset timing definition above (hospital acquired respiratory viruses, HO-MDRO infections, etc.)
   10. *Only interested in infection not colonization
2. Does it focus on hospitalized or inpatients?
   1. *By default, surgical populations count as “inpatients” for our purposes
   2. Is the population presented mixed (inpatient and outpatient)? Only include if data are presented specifically for the inpatient population.
3. Does the article provide objective data so that patients with differences in race, ethnicity, and/or a SDoH can be compared?
   1. To be included in the statistical evaluation, data must be presented with numerator and denominator

**Exclusion criteria**

1. Wrong patient population
   1. Results summarize outpatients
   2. COVID-19 related manuscripts unless discussion of HO - respiratory virus or COVID
   3. Impact on healthcare workers
2. Wrong outcome
   1. Even if outcomes summarize MDRO or other infection of interest (CLABSI), focus on infections with onset in hospital
      1. For CLABSI example, exclude if focus is on CLABSI incidence among patients on outpatient HD (even if hospitalized as a result of the infection). ***Key = timing of onset***
   2. Similarly, do not include community-acquired (CA)- or healthcare facility-associated (HCFA)-CDI
   3. Exclude if infection onset in nursing home or any other non-acute care hospital setting
   4. HAI defined correctly, but included as part of aggregate outcome (e.g., “complications” or HAC). Specific data on R/Eth/SDoH and the specific HAI not presented as a sub-analysis.
   5. Colonization not infection
3. Wrong setting
   1. Non-US (Allowance for use of databases such as ACS NSQIP if US studies represent >90% of sites)
   2. Non-English language
4. Wrong study design
   1. Not original human research - Commentary, invited article, letter
   2. Basic or translational science articles
   3. Animal models

**If article should be excluded, choose reason for exclusion from drop-down menu.**

**If article should be included, add TAG(s)* for race/ethnicity/SDoH category(s) AND HAI. If study involved use of questionnaire or survey to identify SDoH, please tag. If SDoH, choose tag from six categories below:**

| **Economic Stability** | **Neighborhood**  **Environment** | **Education** | **Food** | **Community and Social Context** | **Healthcare System** |
| --- | --- | --- | --- | --- | --- |
| Income | Geography/Zip Code | Early childhood education | Hunger | Social integration | Health coverage |
| Medical Bills | Environment/ Pollution | Higher education | Access to healthy options | Support Systems | Provider availability |
| Socioeconomic Status | Safety | Literacy | Obesity | Community engagement | Provider linguistic and cultural competency |
| Debt | Park | Vocational training | Malnutrition | Age/Sex/gender | Veterans/ VA |
| Employment | Walkability |  |  | Stress | Quality of Care |
| Support | Playgrounds |  |  | Discrimination | Insured/  insurance |
| Expenses | Transportation |  |  | Sexuality  Sexual orientation |  |
|  |  |  |  | Abuse/neglect |  |

**Note that TAGs carry forward from T/A review; alter as applicable based on full text review*

1. **Risk of Bias Assessment**

**JBI Critical Appraisal Tools utilized for the study available at this link:** [**https://jbi.global/critical-appraisal-tools**](https://jbi.global/critical-appraisal-tools%20)

**S3.** Quality of evidence assessment of included studies using the Joanna Briggs Institute (JBI) Critical Appraisal Checklist.

| **Source** | **1. Were the two groups similar and recruited from the same population?** | **2.Were the exposures measured similarly to assign people to both exposed and unexposed groups?** | **3.Was the exposure measured in a valid and reliable way?** | **4.Were confounding factors identified?** | **5.Were strategies to deal with confounding factors stated?** | **6.Were the groups/participants free of the outcome at the start of the study?** | **7.Were the outcomes measured in a valid and reliable way?** | **8. Was the follow up time reported and sufficient to be long enough for outcomes to occur?** | **9.Was follow up complete, and if not, were the reasons to loss to follow up described and explored?** | **10.Were strategies to address incomplete follow up utilized?** | **11.Was statistical analysis appropriate?** |
| --- | --- | --- | --- | --- | --- | --- | --- | --- | --- | --- | --- |
| Allareddy (2014) | yes | yes | yes | yes | yes | unclear | yes | yes | yes | no | yes |
| Argamany (2016) | unclear | yes | yes | yes | yes | yes | yes | yes | not applicable | not applicable | yes |
| Arsoniadis (2017) | yes | yes | yes | no | no | yes | yes | yes | unclear | unclear | no |
| Bakullari (2014) | yes | yes | yes | yes | yes | yes | yes | yes | not applicable | not applicable | yes |
| Blum (2013) | yes | yes | yes | yes | yes | yes | yes | yes | not applicable | not applicable | yes |
| Browne (2014) | yes | yes | yes | yes | yes | yes | yes | yes | not applicable | not applicable | yes |
| Bucher (2011) | yes | yes | yes | yes | yes | yes | yes | yes | not applicable | not applicable | yes |
| Burke (2009) | yes | yes | yes | yes | yes | yes | yes | yes | yes | yes | yes |
| Chen (2019) | yes | yes | yes | yes | yes | yes | yes | yes | unclear | no | yes |
| Edwards (2020) | yes | yes | yes | yes | yes | yes | yes | unclear | yes | yes | yes |
| Egorova (2015) | yes | yes | yes | yes | yes | yes | yes | yes | yes | yes | yes |
| Fargen (2015) | yes | yes | yes | yes | yes | yes | yes | yes | not applicable | not applicable | yes |
| Gouel-Cheron (2022) | yes | yes | yes | yes | yes | yes | yes | yes | not applicable | not applicable | yes |
| Gualandi (2018) | yes | yes | yes | yes | yes | unclear | yes | yes | yes | not applicable | yes |
| Hogle (2014) | yes | yes | yes | unclear | no | yes | yes | yes | yes | yes | yes |
| Jeon (2014) | yes | yes | yes | yes | yes | unclear | yes | yes | not applicable | not applicable | yes |
| Liang (2013) | yes | yes | yes | yes | yes | yes | yes | yes | not applicable | not applicable | yes |
| Liu (2020) | yes | yes | yes | yes | yes | yes | yes | yes | yes | not applicable | yes |
| Milstone (2011) | yes | yes | yes | yes | yes | not applicable | yes | yes | yes | yes | yes |
| Myssiorek (2018) | yes | yes | yes | yes | yes | yes | yes | yes | no | no | yes |
| Namba (2012) | yes | yes | yes | yes | yes | yes | yes | yes | not applicable | not applicable | yes |
| Namba (2013) | yes | yes | yes | yes | yes | yes | yes | yes | not applicable | not applicable | yes |
| Poultsides (2013) | yes | yes | yes | yes | yes | yes | yes | yes | not applicable | not applicable | yes |
| Qi (2019) | yes | yes | yes | yes | yes | yes | yes | yes | not applicable | not applicable | yes |
| Richards (2014) | yes | yes | yes | yes | yes | yes | yes | yes | unclear | unclear | yes |
| Ricciardi (2008) | yes | yes | yes | yes | yes | yes | yes | yes | not applicable | not applicable | yes |
| Rosenblatt (2019) | yes | yes | yes | yes | yes | yes | yes | yes | yes | not applicable | yes |
| Simon (2009) | yes | yes | yes | yes | yes | yes | yes | yes | not applicable | not applicable | yes |
| Singh (2020) | yes | yes | yes | yes | yes | yes | yes | yes | not applicable | not applicable | yes |
| Singh (2022) | yes | yes | yes | yes | yes | yes | yes | yes | yes | not applicable | yes |
| Snyder (2020) | yes | yes | yes | yes | yes | yes | yes | not applicable | not applicable | not applicable | yes |
| Theiss (2022) | yes | yes | yes | yes | yes | yes | yes | unclear | no | no | yes |
| Tilton (2019) | yes | yes | yes | yes | unclear | yes | unclear | yes | yes | yes | yes |
| Trilles (2022) | yes | yes | yes | yes | yes | yes | yes | unclear | no | no | yes |
| Vader (2021) | yes | yes | yes | unclear | yes | yes | yes | yes | yes | unclear | yes |
| Wang (2022) | yes | yes | yes | yes | yes | yes | yes | unclear | no | no | yes |
| Willer (2022) | yes | yes | yes | yes | yes | yes | yes | not applicable | not applicable | not applicable | yes |
| Zarzaur (2013) | yes | yes | yes | yes | yes | yes | yes | yes | not applicable | not applicable | yes |
| Zhao (2014) | yes | yes | yes | yes | yes | yes | yes | yes | unclear | unclear | yes |

**S4: MOOSE Checklist**

**See separate PDF**

**S5: Study results by HAI and SDOH element**

|  | **SSI** | **HO-BSI/CLABSI** | **HO-CDI** | **VAP** | **CAUTI** | **MRSA** |
| --- | --- | --- | --- | --- | --- | --- |
| **Race and ethnicity** | ↑risk: Black (n=6),  ↔risk: Black(n=9), Hispanic (n=8)  ↓risk: Black (n=3)  Hispanic (n=2) | ↑risk: Black (n=4), Hispanic (n=3)  ↓risk: Hispanic (n=1)  ↔risk: Hispanic (n=2)  Black (n=1) | ↑risk: Black (n=1),  ↔risk: Black (n=1) Hispanic (n=1) | ↑risk: Black (n=1),  ↓risk: nonwhite race (n=1) |  | ↑risk: Black (n=3), Native American (n=1)  ↓risk: Hispanic (n=1), Asian (n=1) |
| **Sex** | ↑risk: Male (n=4)  Female (n=2)  ↔risk: Sex (n=2) | ↓risk: Female (n=2) | ↑risk: Female (n=1)  ↔risk: Sex (n = 1) | ↓risk: Female (n=1) |  | ↓risk: Female (n=2) |
| **Age** | ↑risk: Neonates (n=2)  Age >80 (n=2)  Younger age (n=1)  ↓risk: Age >40 (n=2)  ↔risk: Age(n=2), | ↑risk: age 6-12 months (n=1), younger age (n=1) | ↑risk: age > 70 (n=1) |  |  | ↓risk: Increasing age (n=1) |
| **Economic stability** | ↑risk: low income (n=1) | ↔risk: median income (n=1) |  | ↔risk: median income of home zip code (n=1) |  |  |
| **Neighborhood & Physical Environment** |  | ↑risk: suburban area (n=1)  Southern US (n=1)  ↔risk: other US regions, rural or metro areas (n=1) | ↓risk: rural hospital (n=1)  ↔risk: census tract (n=1) | Variable VAP rates seen by zip code (n=1) |  |  |
| **Education** | ↑risk: low health literacy (n=1) |  |  |  |  |  |
| **Food and Nutrition** | ↑risk: obesity (n=3) |  |  |  |  |  |
| **Community and Social Context** |  |  |  |  |  |  |
| **Health System** | ↑risk: Medicaid (n=3)  Medicare (n=1)  Medicare Advantage (ref Medicare) (n=1)  ↔risk: Medicare insurance (n=1) | ↑risk: Public insurance (n=2) | ↑risk: Public insurance (n=2) |  | ↑risk: Public insurance (n=1) | ↑risk: Public insurance (n=1)  ↓risk: Insured (n=1) |

Note: White race is the reference group for all racial comparisons. Private insurance is the reference group for all insurance comparisons unless otherwise specified.

**S6: Summary of Meta-Analyses for which sufficient data was available with common HAI, risk factor and reference category.**

|  | Black/White | Hispanic/White | Male/Female | Medicare/Private |
| --- | --- | --- | --- | --- |
| CLABSI | X | X |  |  |
| SSI | X | X | X | X |
| HO-CDI | X | X |  |  |

**S7: Sex and Surgical Site Infection**

Individual study details are available in Table 1. Surgeries were diverse including total knee, hip, or shoulder arthroplasty, hysterectomy, bariatric surgery, colon surgery, genital reconstruction and other general surgeries.

**S8: Insurance Payors and Surgical Site Infection**

Individual study details are available in Table 1. Surgeries were diverse including total knee, hip, or shoulder arthroplasty, colectomy, hysterectomy, and CSF shunt placement.

**S9: Study Data Sources**

| **Manuscript**  **First author (year)** | **Source of exposure variables of interest** | **Description of data collection and validation** |
| --- | --- | --- |
| **National Administrative Databases** | | |
| Allareddy (2015) | US Nationwide Inpatient Sample | Retrospective; database composed of data derived from EHRs and billing data from participating sites. Per SOP notes, “reporting of information on race and ethnicity can vary by hospital.” <https://hcup-us.ahrq.gov/db/vars/race/nisnote.jsp> |
| Browne (2014) |  |  |
| Egorova (2015) |  |  |
| Fargen (2015) |  |  |
| Poultsides (2013) |  |  |
| Ricciardi (2008) |  |  |
| Rosenblatt (2019) |  |  |
| Singh (2020) |  |  |
| Argamany (2016) | CDC National Hospital Discharge Survey | Retrospective; data compiled from two sources: 1) manual sample selection and transcription of information from hospital records and 2) via an automated system in which CDC purchases electronic files of electronic data from commercial organizations (<https://www.cdc.gov/nchs/nhds/nhds_collection.htm>). Validation procedures have been performed to ensure data transmitted to CDC is reflective of data in health records but not of validity of primary data. |
| Arsoniadis (2017) | ACS-NSQIP | Retrospective; data abstracted and entered into database by “trained surgical nurse reviewers” (<https://www.facs.org/media/yaol5yoj/nsqip_puf_userguide_2020.pdf>). Data largely derived from local health records. |
| Chen (2019) |  |  |
| Myssiorek (2018) |  |  |
| Singh (2022) |  |  |
| Trilles (2022) |  |  |
| Bakullari (2014) | Medicare Patient Safety Monitoring System | Retrospective; data compiled from annual random national sampling of hospital records from more than 40,000 Medicare inpatient discharges ([www.ahrq.gov/downloads/pub/advances/vol2/Hunt.pdf](http://www.ahrq.gov/downloads/pub/advances/vol2/Hunt.pdf)). Validation steps in place for abstraction into database but no validation of primary data. |
| Snyder (2020) | Healthcare Cost and Utilization Project (HCUP) Kid's Inpatient Database | Retrospective; see US Nationwide Inpatient Sample, data limited to pediatric inpatient admissions. |
| Wang (2022) | Premier National Database | Retrospective; data derived from electronic health records and billing data |
| **State-Level Databases** | | |
| Blum (2013) | Pennsylvania Health Care Cost Containment Council database | Retrospective; state legislature-mandated database that includes inpatient hospital discharge records |
| Liu (2020) | California Perinatal Quality Care Collaborative (CPQCC) | Retrospective; data definitions state maternal ethnicity and race “should be completed by or with direct assistance of the informant” (<https://cpqcc.org/files/2025_NICU_Data_Manual_of_Definitions.pdf>) |
| Qi (2019) | State Inpatient Databases for AZ, FL, IA, MA, MD, NY, VT | Retrospective; see US Nationwide Inpatient Sample |
| **Multisite Quality Improvement Databases** | | |
| Edwards (2020) | Metabolic and Bariatric Surgery Accreditation and Quality Improvement Program Participant Use File Database | Retrospective; data collected by trained clinical reviewers at each bariatric center with planned audits based on NSQIP protocols. Data typically extracted from participating site EHR ([www.facs.org/media/fdejfgqf/mbsaqip-puf-user-guide-2023.pdf](http://www.facs.org/media/fdejfgqf/mbsaqip-puf-user-guide-2023.pdf)). |
| Gouel-Cheron (2022) | Cerner Healthfacts Database | Retrospective; deidentified clinical data repository from US hospital using Cerner EHR, including EHR-based clinical and administrative data. |
| Namba (2012) | Kaiser Permanente Total Joint Replacement and Implant Registries | Retrospective; data collected on forms integrated into clinical workflow to produce standardized progress notes for EHR documentation at the time of the procedure, supplemented by additional data from EHR and administration claims. |
| Namba (2013) |  |  |
| Richards (2014) |  |  |
| Gualandi (2018) | CDC Emerging Infections Program dataset | Retrospective; dataset developed through collaboration between CDC, state health departments, and academic partners with data collected via standardized case reports. Race included as documented in the EHR. |
| Simon (2009) | Pediatric Health Information System database | Retrospective; data compiled from deidentified discharge-level data from >40 free-standing tertiary care hospitals affiliated with the Children’s Hospital Association. Site-submitted data undergoes consistency reviews and quality audits. No specific mention of validation of primary data. |
| Willer (2022) |  |  |
| Study Hospital EHR | | |
| Bucher (2011) | Study hospital EHR | Retrospective; data extracted from EHR |
| Burke (2009) | Study hospital EHR | Retrospective; data extracted from medical record |
| Hogle (2014) | Study hospital EHR | Retrospective; data extracted from EHR |
| Jeon (2014) | Study hospital EHR | Retrospective; data extracted from EHR |
| Liang (2013) | Study hospital EHR | Retrospective; data extracted from medical record |
| Milstone (2011) | Study hospital EHR | Retrospective; data extracted from medical record |
| Theiss (2022) | Study hospital EHR | Retrospective; data extracted from EHR |
| Tilton (2019) | Study health system EHR | Retrospective; data extracted from EHR |
| Vader (2021) | Study hospital EHR | Retrospective; data extracted from EHR |
| Zarzaur (2013) | Study hospital EHR | Retrospective; data extracted from medical record |
| Zhao (2014) | Study hospital EHR | Retrospective; data extracted from medical record |
